# Platelet-Derived Extracellular Vesicles Increase Col8a1 Secretion and Vascular Stiffness in Intimal Injury

**DOI:** 10.3389/fcell.2021.641763

**Published:** 2021-03-02

**Authors:** Han Bao, Zi-Tong Li, Lei-Han Xu, Tong-Yue Su, Yue Han, Min Bao, Ze Liu, Yang-Jing Fan, Yue Lou, Yi Chen, Zong-Lai Jiang, Xiao-Bo Gong, Ying-Xin Qi

**Affiliations:** ^1^Institute of Mechanobiology & Medical Engineering, School of Life Sciences & Biotechnology, Shanghai Jiao Tong University, Shanghai, China; ^2^Key Laboratory of Hydrodynamics (Ministry of Education), Department of Engineering Mechanics, School of Naval Architecture, Ocean and Civil Engineering, Shanghai Jiao Tong University, Shanghai, China; ^3^Key Laboratory for Biomechanics and Mechanobiology of Ministry of Education, School of Biological Science and Medical Engineering, Beihang University, Beijing, China; ^4^Beijing Advanced Innovation Center for Biomedical Engineering, Beihang University, Beijing, China

**Keywords:** intimal injury, vascular stiffness, platelet-derived extracellular vesicles, vascular smooth muscle cell, col8a1

## Abstract

The arterial mechanical microenvironment, including stiffness, is a crucial pathophysiological feature of vascular remodeling, such as neointimal hyperplasia after carotid endarterectomy and balloon dilatation surgeries. In this study, we examined changes in neointimal stiffness in a Sprague-Dawley rat carotid artery intimal injury model and revealed that extracellular matrix (ECM) secretion and vascular stiffness were increased. Once the endothelial layer is damaged *in vivo*, activated platelets adhere to the intima and may secrete platelet-derived extracellular vesicles (pEVs) and communicate with vascular smooth muscle cells (VSMCs). *In vitro*, pEVs stimulated VSMCs to promote collagen secretion and cell adhesion. MRNA sequencing analysis of a carotid artery intimal injury model showed that ECM factors, including col8a1, col8a2, col12a1, and elastin, were upregulated. Subsequently, ingenuity pathway analysis (IPA) was used to examine the possible signaling pathways involved in the formation of ECM, of which the Akt pathway played a central role. *In vitro*, pEVs activated Akt signaling through the PIP_3_ pathway and induced the production of Col8a1. MicroRNA (miR) sequencing of pEVs released from activated platelets revealed that 14 of the top 30 miRs in pEVs targeted PTEN, which could promote the activation of the Akt pathway. Further research showed that the most abundant miR targeting PTEN was miR-92a-3p, which promoted Col8a1 expression. Interestingly, knockdown of Col8a1 expression *in vivo* abrogated the increase in carotid artery stiffness and simultaneously increased the degree of neointimal hyperplasia. Our results revealed that pEVs may deliver miR-92a-3p to VSMCs to induce the production and secretion of Col8a1 *via* the PTEN/PIP3/Akt pathway, subsequently increasing vascular stiffness. Therefore, pEVs and key molecules may be potential therapeutic targets for treating neointimal hyperplasia.

## Introduction

Extracellular matrix (ECM) stiffness can directly influence many aspects of physiological and pathological processes, including intestine and lung morphogenesis (Rabelink et al., 2017), blood cell development (Leiva et al., 2018), tumor invasion (Schedin and Keely, 2011) and vascular aging (Lacolley et al., 2017). At the cellular level, the stiffness of the extracellular environment plays roles in orienting cell division, directing cell migration, and driving cell differentiation (Handorf et al., 2015). In the cardiovascular system, arterial stiffness associated with cardiovascular diseases has been explored extensively and has been indicated to have a huge impact (Harvey et al., 2016). In hypertension, ECM remodeling, including increasing collagen deposition, cross-linking collagen, and breaking down elastic laminae, affects arterial stiffness and promotes the proliferation of endothelial cells (ECs) and vascular smooth muscle cells (VSMCs) (Thenappan et al., 2018). In addition, in atherosclerosis, the elastic modulus of the thoracic aorta reaches 15 kPa in comparison with 5 kPa in normal mice and increases VSMC proliferation, apoptosis and osteochondrogenic transformation (Xie et al., 2018). Moreover, arterial stiffness could become an important marker to characterize vascular damage (Duprez and Cohn, 2007).

Injury of the arterial endothelial lamina, also called intimal injury, usually occurs after carotid endarterectomy (Budincevic et al., 2015), peripheral artery brachytherapy (Fokkema et al., 2012), stent placement for arterial occlusive disease (Dangas and Kuepper, 2002) and balloon dilatation surgeries (Quencer and Arici, 2015). After intimal injury, VSMC accumulation and extracellular matrix deposition result in intimal hyperplasia and vessel or stent occlusion (Cai et al., 2015). During this process, VSMCs change from a quiescent contractile phenotype to an active synthetic phenotype, which can migrate, proliferate and secrete ECM (Rudijanto, 2007). Subsequently, ECM remodeling changes the mechanical properties of the artery, including vascular elasticity (Stephan et al., 1997) and stiffness (Jaminon et al., 2019). For example, in mechanically injured iliac artery segments in rabbits, collagen types I and III and vascular stiffness were increased, which could induce plaque vulnerability (Kanshana et al., 2018). Although ECM remodeling and vascular stiffness play important roles in regulating intimal injury and intimal hyperplasia, the changes in ECM components and the factors regulating these changes are not fully understood.

There are many factors that can cause VSMC phenotypic transformation to remodel the ECM, such as multiple growth factors (Rudijanto, 2007; Guo et al., 2014), noncoding RNAs (Zeng et al., 2019), lipoproteins (Greig et al., 2015) and chemical compounds (Guo et al., 2019). In addition, platelets, a cellular debris shed by megakaryocytes, was originally thought to be components of coagulation, and now have been proven to play crucial roles in VSMC dysfunction. During intimal injury, platelets are activated, adhere to the intima and participate in intercellular communication with VSMCs (Pang et al., 2019; Zeng et al., 2019). Activated platelets secrete multiple agents, such as thromboxane and PDGF (Pang et al., 2019), leading to the migration and proliferation of VSMCs and promoting the formation of plaques. In recent years, research has shown that platelet-derived extracellular vesicles (pEVs), which are released by activated platelets, can transport molecules and participate in intercellular communication (Laffont et al., 2013). For example, pEVs containing serotonin and TXA2 could induce rabbit VSMC proliferation at the sites of vascular injury (Pakala, 2004).

Extracellular vesicles (EVs), which range in size from 40 nm to 5 μm, include small EVs (exosomes, arrestin-domain-containing protein 1-mediated microvesicles, etc.) and large EVs (microvesicles, apoptotic bodies, etc.) (Jeppesen et al., 2019). Previous studies showed that there were many bioactive molecules delivered by pEVs, including lipids, proteins, nucleic acids, and organelles involved in numerous biological processes (Puhm et al., 2020). For example, pEV-derived TGF-β can induce the differentiation of naïve CD4^+^ T cells into Foxp3^+^ regulatory T cells, which can influence T lymphocytes that are recruited to atherosclerotic lesions (Vajen et al., 2015). In addition, pEVs can deliver small noncoding RNAs, such as miR-223, to ECs and repress the expression of target mRNAs, such as FBXW7 and EFNA1 (Laffont et al., 2013). Although pEVs can participate in the regulation of VSMC proliferation (Pakala, 2004) and migration (Shan et al., 2015) through the delivery of a variety of molecules, it is still unclear whether pEVs can affect VSMC-induced ECM remodeling and arterial stiffness in the context of intimal injury.

In the present study, we examined vascular stiffness in intimal injury and the regulatory mechanisms of pEVs in ECM remodeling mediated by VSMCs. This study may provide new insight into the changes in vascular stiffness in intimal injury and may provide novel mechanoresponsive targets for the maintenance of vascular homeostasis.

## Materials and Methods

The main methods are described in the text, and additional methods are detailed in the Supporting Information (Supplementary Materials and Methods).

### Rat Carotid Artery Intimal Injury Model

The animal care and experimental protocols were conducted in accordance with the Animal Management Rules of China (55, 2001, Ministry of Health, China), and the study was approved by the Animal Research Committee of Shanghai Jiao Tong University.

Male Sprague-Dawley (SD) rats with an average weight of 400 g were anesthetized with isoflurane inhalation (MATRX VIP 3000, United States). The left carotid arteries were exposed, and a percutaneous transluminal angioplasty balloon dilatation catheter (2 F, 0.67 mm, Edwards Lifesciences, United States) was used to establish vascular intimal injury (Raugi et al., 1990). The arteries were then harvested after 2 weeks, and the undamaged right carotid artery served as the self-control.

### Immunofluorescence Staining

The carotid arteries samples were fixed in 4% paraformaldehyde, dehydrated in 30% sucrose solution, and then cut into 6-μm sections. The frozen sections were washed three times with PBS, permeabilized with 0.3% Triton X-100 for 30 min, and immersed in a solution of 10% goat serum for 30 min at room temperature to block nonspecific binding. Subsequently, the sections were incubated with the primary antibodies against Col8a1 (1:400, Proteintech Group, United States), Col8a2 (1:400, Abcepta, United States), SMA (1:500, Invitrogen, United States), vWF (1:500, Cell Signaling Technology, United States), CD41 (1:500, Cell Signaling Technology, United States) at 4°C overnight. After incubated with secondary antibody (1:1000. Abcam, United Kingdom) for 2 h, DAPI was used for nuclei staining for 15 min at room temperature. Staining at the cellular level, paraformaldehyde was used to fix VSMCs for 30 min and then followed the above method. Photographs were taken by confocal microscopy (LV1000; Olympus).

### FISH Analysis

Six-μm frozen-sections of carotid artery samples were treated with 0.3% H_2_O_2_ to block endogenous peroxide activity and proteinase K (5 mg⋅mL^–1^) for permeabilization. The samples were then hybridized with a miR-92a-3p biotinylated probe or a NC probe (Shanghai GenePharma, China) (200 nM) overnight at 56°C. The FISH signals were amplified with Tyramide SuperBoost Kits (Thermo Fisher Scientific, United States) followed by an Alexa Fluor Tyramide Kit (Thermo Fisher Scientific, United States), and photographed under confocal microscopy (LV1000; Olympus). The sequences of the RNA oligos were listed in Supplementary Table 1.

### Ultrasound Imaging

Multi-mode Ultrasound Imaging System (Fujifilm VisualSonics, United States) was used to detect the change in arterial diameter (%) of the carotid artery in rats after intimal injury. SD rats were anesthetized with isoflurane (MATRX VIP 3000, United States) and signals were collected using 20 MHz MX Series transducer (MX250S) in “M-Mode”. Data analysis were performed on FUJIFILM VisualSonics Measurement software.

### Vascular Stiffness Measurement

Piuma Nanoindenter (Optics11, Netherlands) was used to detect the stiffness of the carotid artery after intimal injury (Xie et al., 2018). A probe with a 0.49 N⋅m^–1^ spring constant and a 31 μm spherical indentation radius was used. All measurements were performed with the carotid artery flattened onto the bottom of a dish and submerged in PBS at room temperature. The indents were depth controlled (10 μm), and the loading and unloading period was set to be 2 s. Based on the load-displacement curves, the Young’s modulus was calculated using the Hertz spherical indentation model in Piuma Software (version: V3.3.0). For vascular stiffness analysis, 5-10 measurements were made on each carotid artery.

### Nanoparticle Tracking Analysis

Nanoparticle tracking analysis was used to analyze the number and diameter of pEVs (Szatanek et al., 2017). The obtained pEVs were diluted with PBS and loaded into the NanoSight module (NanoSight NS300, United Kingdom) for measurement. The module was washed with PBS after each measurement.

### Transcriptome Sequencing and miR Sequencing

The intimal injury arteries harvested after 2 weeks, and the undamaged right carotid artery served as the self-control, then performed transcriptome sequencing (GEO accession numbers: GSE164050). And the miR sequencing used by activated platelets and platelets released pEVs. Differentially expressed mRNAs or miRs were analyzed utilizing DESeq with the following criteria: fold change > 2 and false discovery rate (FDR) < 0.05. Afterwards, the ClustVis. web tool^1^ was used to upload raw data and create heatmaps.

### Ingenuity Pathway Analysis

The possible biological processes and functional classifications were obtained with Ingenuity Pathway Analysis (IPA) software^2^ (Content version: 57662101, Qiagen, Germany). “Formation of extracelluar matirx” related genes in “Diseases and Functions model” were fist selected based on transcriptome sequencing data. Besides, the most abundant 30 miRs expressed in pEVs were uploaded into IPA to analyze their downstream target genes and main functions involved in. IPA integrated the available knowledge on genes, drugs, chemicals, protein families, processes, and pathways based on the interactions and functions derived from the Ingenuity Pathways Knowledge Database Literature, and understands the complex biological and chemical systems at the core of life science research based on lectures or predicated analysis (Dai et al., 2009).

### Stimulation of VSMCs With LY294002

LY294002 (10 μM), a highly selective inhibitor of phosphatidylinositol 3 (PI3) kinase, was preincubated with VSMCs for 1 h before adding pEVs. The same volume of DMSO was used as control.

### PIP_3_ ELISA

The concentration of PIP_3_ in VSMCs was determined by ELISA using a rat PIP_3_ ELISA kit (Shanghai FanTai Biotechnology, China). The kit used bi-antibody sandwich method to determine the level of PIP_3_. First of all, the sample added to the microwells of the coated PIP_3_ monoclonal antibody, and then it is combined with HRP-labeled PIP_3_ antibody to form an antibody-antigen-enzyme-labeled antibody complex. After 3 times washing by PBS, the substrate TMB and acid were added for chromogenic reaction. The absorbance (OD value) was measured with the microplate reader (Bio-Rad 680, Bio-Rad, United States) at a wavelength of 450 nm.

### Dual Luciferase Reporter Assay

The 3′ untranslated regions (UTRs) of PTEN including the predicted miR-92a-3p binding sequences, and the mutation segment were all obtained by gene synthesis. The segments were inserted into the downstream of the luciferase reporter gene (psiCheck-2, Promega, United States), respectively. To determine the suppressing efficiency of miR-92a-3p, HEK-293T cells were transfected with the reporter plasmid or the mutated vectors together with miR-92a-3p mimic or NC. Twenty-four h later, firefly and renilla luciferase activities were measured consecutively using a dual luciferase reporter assay system (Promega, United States).

### Local Injection of Col8a1 SiRNA

After carotid artery intimal injury surgery, the rats were randomly assigned to two groups: subcutaneous injections of 300 μl of anti-col8a1 siRNA (1 μM) or negative control (Pillé et al., 2005). Subcutaneous injections were repeated every 2 days for a total of 2 weeks.

### Statistical Analysis

Each experiment was performed at least in quadruplicate of biological replicates. Statistical analysis was performed and figures were prepared with the GraphPad Prism 6.0 (GraphPad Software, CA, United States). All values are expressed as the mean ± SD. The Gaussian distribution of values was analyzed by the Kolmogorov-Smirnov test. A paired *t*-test was used for paired data with a Gaussian distribution; A Wilcoxon matched-pairs signed rank test was used for paired data that lacked a Gaussian distribution or a sample size that was less than 5. In addition, the Friedman test was used for multiple comparisons with a single reference group whose sample size was less than 5. An unpaired *t*-test was used for unpaired data with a Gaussian distribution. Differences with values of *P* < 0.05 were regarded as statistically significant.

## Results

### Intimal Injury Increases Vascular Stiffness and Promotes Collagen Accumulation

**FIGURE 1:**
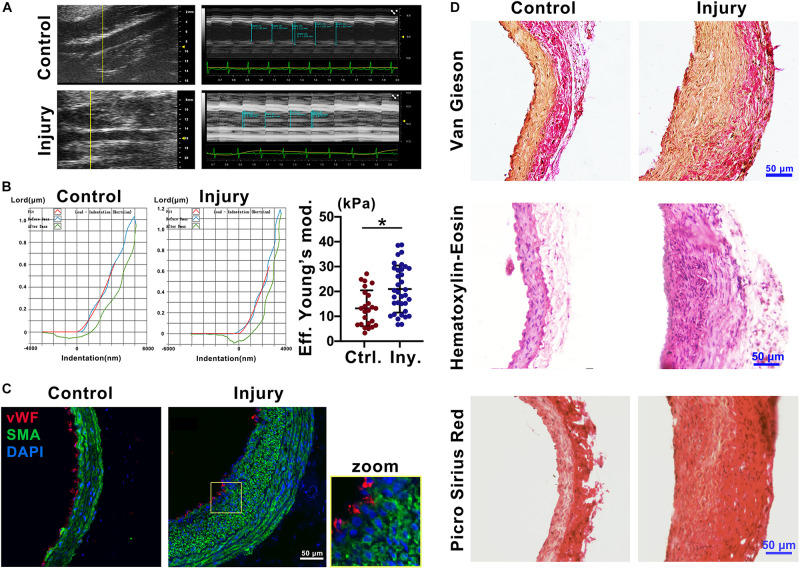
Intimal injury promoted neointimal hyperplasia and increased vascular stiffness. **(A)** Ultrasound imaging was used to measure the change in arterial diameter (%). At 2 weeks after intimal injury surgery, the change in arterial diameter was significantly decreased. **(B)** The Piuma nanoindenter was used to determine vascular stiffness, and the stiffness of the carotid artery increased from an average of 13.45 kPa to an average of 20.98 kPa after intimal injury surgery. **(C)** Immunofluorescence was used to determine the type of cells in areas of neointimal hyperplasia, and most of the hyperplasia involved VSMCs. Green indicates SMA staining (VSMCs), red indicates vWF staining (ECs), and nuclear staining is shown in blue by DAPI (bar = 50 μm). **(D)** Elastin-van Gieson, hematoxylin-eosin (HE), and picrosirius red staining revealed that the area of neointimal hyperplasia was significantly thickened and collagen accumulated 2 weeks after intimal injury compared with those of the contralateral common carotid artery (control) (bar = 50 μm). The values are shown as the mean ± SD, *P < 0.05 vs. control (*n* = 4 biological replicates).

To explore the mechanical properties of the carotid artery after injury, we used ultrasound imaging to measure the deformation of the carotid artery following cardiac pulsation (Figure 1A) and a Piuma Nanoindenter to measure the stiffness of the carotid artery (Figure 1B) at 2 weeks after injury. Compared with that of the self-contralateral common carotid artery, the change in arterial diameter (%) (Safar et al., 1981) at 2 weeks after intimal injury surgery was significantly decreased (Figure 1A). Moreover, compared with that of the control, the stiffness of the carotid artery increased from an average of 13.45 kPa to an average of 20.98 kPa (Figure 1B).

To address the progression of vascular remodeling after intimal injury, elastin-van Gieson, hematoxylin-eosin (HE), and picrosirius red staining were used to examine the neointima, vascular morphology and collagen levels in the common carotid artery at 2 weeks after injury (Figure 1D). Compared with that of the self-contralateral common carotid artery, the neointima and VSMCs were significantly thickened and increased in the injured carotid artery (Figures 1C,D). Collagen accumulated in the vessel wall, especially in areas of neointimal hyperplasia (Figure 1D). These results suggested that in the intimal injury model, the stiffness of the injured vessel was increased at approximately 2 weeks. The main component in areas of hyperplasia was VSMCs, and there was a large amount of collagen in the neointima, which may affect the mechanical properties of the injured artery.

### pEVs Interact With VSMCs to Promote the Secretion of Collagen

**FIGURE 2:**
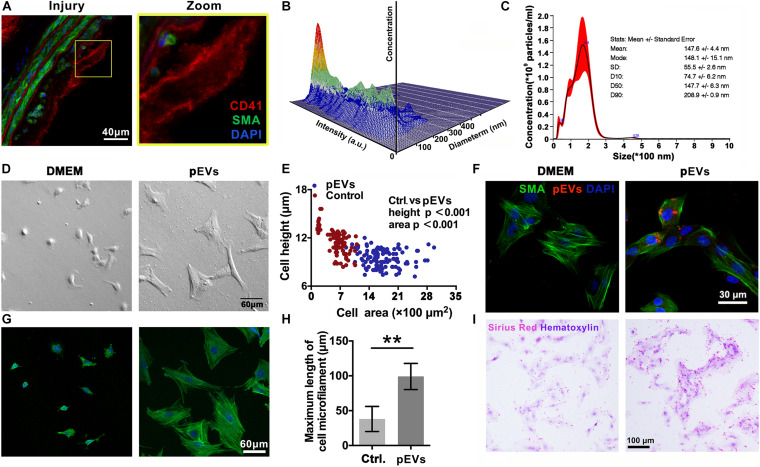
pEVs interacted with VSMCs to promote collagen secretion. **(A)** Immunofluorescence staining was used to examine the adhesion of VSMCs *in situ* compared with that in the control group. Green indicates SMA staining (VSMCs), red indicates CD41 staining (pEVs), and nuclear staining is shown in blue by DAPI (bar = 40 μm). **(B,C)** Nanoparticle tracking analysis (NTA) was used to examine the concentration and size distribution of the pEVs. The results revealed that most of the pEVs were between 100 and 300 nm in size. **(D,E)** Cell morphological analysis revealed the adhesion of VSMCs to glass culture dishes after pEV stimulation. The area and height of cell adhesion were measured by the confocal microscope. After pEV stimulation, more VSMCs adhered and spread on the surface of the glass plate compared with the control (bar = 60 μm). **(F)** pEVs significantly adhered to VSMCs at 1 h. Red fluorescence shows the pEV membranes, which were labeled with the fluorescent cell linker PKH26, green fluorescence shows SMA staining (VSMCs), and blue fluorescence shows DAPI-labeled nuclei (bar = 30 μm). **(G,H)** Microtubule staining showed changes in microtubule length, and more bundles of long microtubules were present in the VSMCs stimulated by pEVs than in unstimulated cells (bar = 60 μm). **(I)** Picrosirius red staining was used to analyze whether VSMC collagen secretion changed after pEV stimulation. Compared with unstimulated VSMCs, VSMCs stimulated with pEVs secreted more collagen (bar = 100 μm). The values are shown as the mean ± SD, *P < 0.05, **P < 0.01 vs. control (*n* = 4 biological replicates).

Immunofluorescence staining revealed that CD41-positive pEVs were closely adjacent to VSMCs in the injured carotid artery *in vivo* (Figure 2A). Then, Nanoparticle Tracking Analysis (NTA) was used to examine the concentration and size distribution of the circulating EVs after intimal injury surgery. The results showed that compared with the control group, the number and size of circulating EVs did not change significantly after intimal injury surgery (Supplementary Figure 1). This may because that the activation of platelets and the release of pEVs mainly occur in the injured area, and cannot significantly affect the number of pEVs in the entire blood.

*In vitro*, Electron microscopy imaging and NTA indicated that the size of most pEVs secreted by activated platelets was between 100 to 200 nm (Figures 2B,C and Supplementary Figure 2). Moreover, nanoparticle tracking analysis (NTA) indicated that the size a peak was at 164 nm. In addition, there was a small peak at 36 nm, indicating that there might be a small amount of exosomes in the extracted pEVs (Figures 2B,C).

To investigate the adhesion of pEVs to VSMCs *in vitro*, pEVs were labeled with the red fluorescent cell linker PKH26. The immunofluorescence staining results showed that PKH26-positive pEVs adhered to VSMCs after pEV stimulation for 1 h (Figure 2F). When pEVs and newly digested and suspended VSMCs were mixed and seeded on an uncoated glass plate, more VSMCs adhered and spread on the glass surface than in the control group (Figures 2D,E), indicating that pEVs promoted VSMC adhesion. In addition, increased bundles of long microtubules were present in VSMCs stimulated by pEVs (Figures 2G,H), which might be because VSMCs secrete large amounts of extracellular matrix (ECM), and VSMCs have improved focal adhesion on a smooth glass plate (Humphries, 2009). Subsequently, picrosirius red staining was used to analyze whether collagen secretion by VSMCs was changed after pEV stimulation. Compared with unstimulated VSMCs, VSMCs stimulated by pEVs secreted more collagen (Figure 2I). These results suggested that during vascular intimal injury, pEVs could interact with VSMCs and cause VSMCs to secrete increased ECM, thereby inducing vascular ECM remodeling. Then, we focused on the ECM, expecially the members of collagen to determine their roles in the process of endometrial injury.

### Intimal Injury and pEVs Upregulate Col8a1 Expression

**FIGURE 3:**
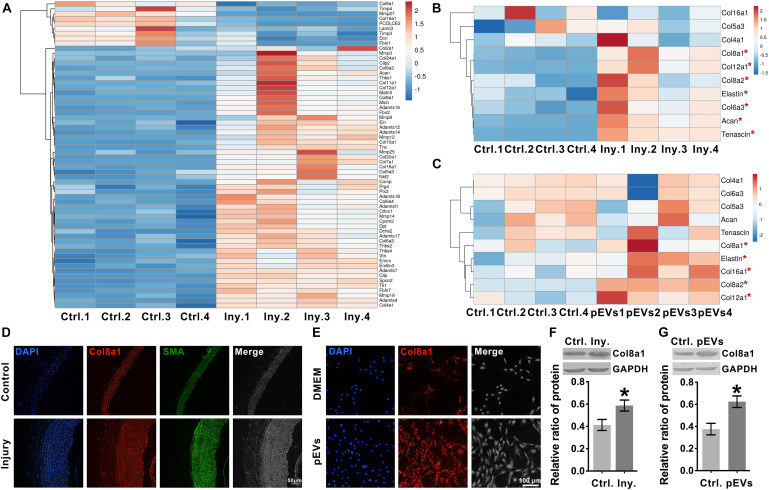
Intimal injury and pEVs regulated the increase in Col8a1 expression. **(A)** Bioinformatics analysis revealed 63 distinct molecules related to ECM remodeling in the intimal injury model by mRNA sequencing. **(B,C)** Real-time RT-PCR was used to measure the expression of the 10 molecules with the highest abundance and differential expression, as determined by mRNA sequencing of extracellular matrix structural components. **(B)** Thee intimal injury group was compared with the self-contralateral common carotid artery, and **(C)** the pEV stimulation group was compared with the control group. **(D,E)** Immunofluorescence staining was used to measure Col8a1 accumulation in response to intimal injury or pEV stimulation *in vitro*. The results showed that in response to either intimal injury **(D)** or pEV stimulation **(E)**, the fluorescence intensity of Col8a1 increased significantly. **(F,G)** Western blotting was used to measure the expression of Col8a1. The results showed that in response to either intimal injury **(F)** or pEV stimulation **(G)**, the expression of Col8a1 increased significantly. The values are shown as the mean ± SD, *P < 0.05 vs. control (*n* = 4 biological replicates).

To explore the ECM and ECM regulatory factors involved in neointimal hyperplasia in an intimal injury model, we used mRNA sequencing to analyze the changes in mRNA expression in the injured carotid artery at 2 weeks after surgery. Among the differentially expressed genes, 237 genes were identified as “extracellular space” by IPA (Supplementary Table 2). Subsequently, we used UniProt^3^ and GO “molecular function” analyses to distinguish these “extracellular space” molecules into the “extracellular matrix structural constituent” and “extracellular matrix organization and metalloendopeptidase activity” categories (Supplementary Table 3). Among them, the “extracellular matrix structural constituent” category contained 20 molecules, and the “extracellular matrix organization and metalloendopeptidase activity” category contained 43 molecules (Supplementary Table 3 and Figure 3A).

We selected the 10 molecules with the highest abundance and greater than 2-fold differential expression in the “extracellular matrix structural constituent” category for subsequent analysis. As shown in Figure 3B, real-time RT-PCR analysis validated that col8a1, col12a1, col8a2, elastin, col6a3, acan and tenascin expression levels were significantly upregulated at 2 weeks after intimal injury surgery compared with those of the self-contralateral common carotid artery. *In vitro*, after pEV stimulation for 12 h, qPCR analysis showed that col8a1, elastin, col16a1, col8a2 and col12a1 expression levels were significantly upregulated compared with those of the control (Figure 3C). The expression levels of col8a1, col8a2, col12a1, and elastin changed in response to both stimuli. Among these molecules, the Col VIII family has been proven to play an important role in changes in the stiffness of atherosclerotic plaques (Merei et al., 2017). Therefore, we performed immunofluorescence and western blotting analyses of Col8a1 and Col8a2.

The immunofluorescence staining results showed that in the neointima after intimal injury *in vivo* and in VSMCs stimulated with pEVs *in vitro*, the fluorescence intensities of Col8a1 and Col8a2 increased significantly (Figures 3D,E and Supplementary Figure 3). Col8a1 was mostly located in the cytoplasm, as shown immunofluorescence staining, and the qPCR and western blotting results revealed that col8a1 mRNA and protein expression also significantly increased (Figures 3F,G). However, Col8a2 was mostly located in the nucleus (Supplementary Figure 3B), which was different from the traditional understanding of VSMCs (Stephan et al., 2004; Adiguzel et al., 2006). These results suggest that pEV-induced VSMCs produce and secrete Col8a1, which may contribute to ECM remodeling during vascular intimal injury.

### PIP_3_/Akt Signaling Regulates the Production of Col8a1

**FIGURE 4:**
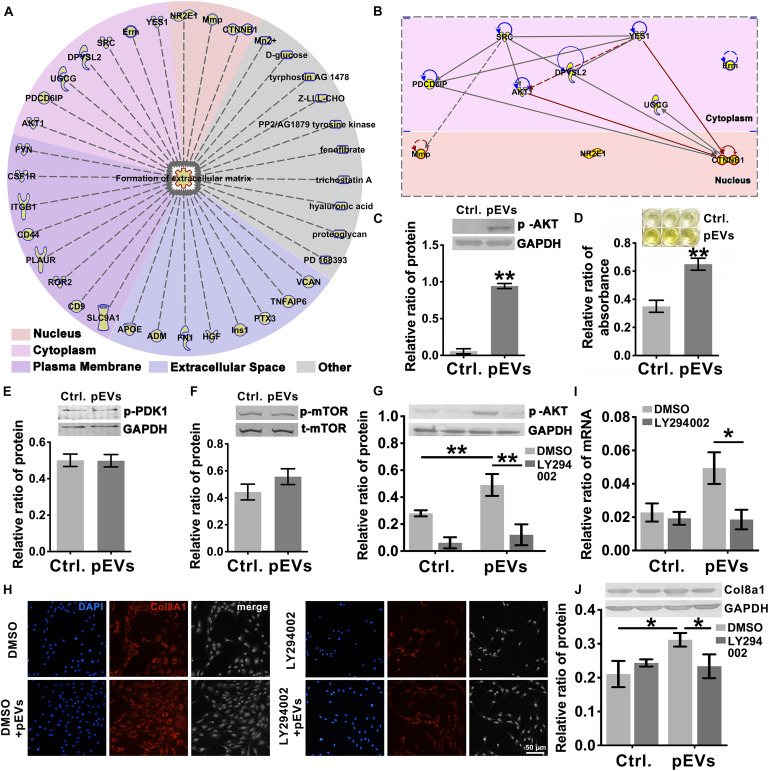
PIP_3_/Akt signaling regulated the production of Col8a1. **(A)** IPA software was used to analyze related molecules that can participate in the regulation of “formation of extracellular matrix” and **(B)** the core signaling pathways in the cytoplasm and nucleus participated in this network. **(C)** Western blotting was used to measure the expression of phosphorylated Akt in VSMCs, and the results showed that Akt was significantly activated after pEV stimulation. **(D–F)** ELISA and western blotting were used to analyze molecules involved in the activation of Akt. Only the level of PIP_3_ increased significantly in VSMCs after pEV stimulation, **(D)** while PDK1 **(E)** and mTORC **(F)** were unchanged. **(G–J)** The specific PIP_3_/Akt signaling inhibitor LY294002 was used to examine the effect of PIP_3_/Akt signaling on the production of Col8a1. **(G)** LY294002 significantly abrogated the expression of p-Akt induced by pEVs and blocked the mRNA **(I)** and protein expression of col8a1 **(H,J)**. The values are shown as the mean ± SD, *P < 0.05, **P < 0.01 vs. control (*n* = 4 biological replicates).

To investigate the molecules transported by pEVs that are involved in regulating the production of Col8a1, IPA software was used to analyze related molecules that may participate in the regulation of “formation of extracellular matrix” (Figure 4A). The results revealed that 36 molecules correlated with the formation of extracellular matrix (Figure 4A and Supplementary Table 4). Among them, we focused on Akt signaling, which is the core signaling pathway that participates in this network (Figure 4B). Western blotting was used to measure the expression of phosphorylated Akt in VSMCs after pEV stimulation, and we found that Akt was significantly activated (Figure 4C). In order to detect whether the Akt carried by pEVs contribute to the Akt changes in VSMCs, which subsequently increased the phosphorylation of Akt, the expression of total Akt in VSMCs after pEVs stimulation was analyzed (Supplementary Figure 4). The results showed that the expression of total Akt was similar between the control group and pEVs stimulation group, which indicated that the increased pAkt may be activated by pEVs but not via the direct delivery.

Since multiple molecules, such as PIP_3_, PDK1 and mTORC2, have been reported to be involved in the activation of Akt (Xu et al., 2015), western blotting and ELISA were further performed (Figures 4D–F). Among these three molecules, only the expression of PIP_3_ increased significantly in VSMCs stimulated with pEVs. The stimulation of pEVs was complicated, and using IPA software the potential interleaving of multiple signal networks which may lead to the activation of Akt and inactivation of PDK1 and mTOR were analyzed (Supplementary Figure 5). Therefore, Akt activation may be due to the activation of upstream PIP_3_ induced by pEVs.

To further verify the effect of PIP_3_/Akt signaling on the production of Col8a1 by VSMCs, cells were pretreated with LY294002, a specific inhibitor of PIP_3_/Akt signaling, for 1 h and then treated with pEVs. LY294002 significantly abrogated the expression level of p-Akt induced by pEVs (Figure 4G). After treatment with pEVs for 12 h, LY294002 also had the same effect on the repression of col8a1 mRNA (Figure 4I). Immunofluorescence staining and western blotting showed that the addition of LY294002 blocked the protein expression of Col8a1 in VSMCs stimulated with pEVs (Figures 4H,J). These results suggested that PIP_3_/Akt signaling participated in the production of Col8a1 by VSMCs stimulated with pEVs.

### miR-92a-3p Delivered by pEVs Induces the Production of Col8a1 by Inhibiting PTEN

**FIGURE 5:**
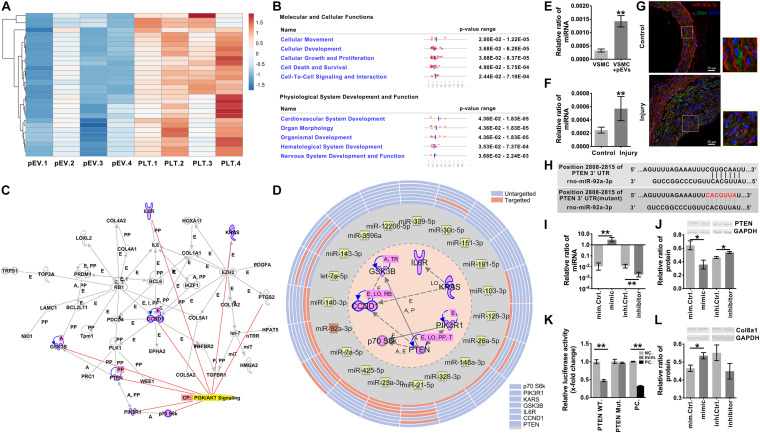
miR-92a-3p upregulated the production of Col8a1 by inhibiting the expression of PTEN. **(A)** mRNA sequencing was used to analyze activated platelets and their secreted pEVs, and the top 30 miRs in pEVs are shown. **(B)** IPA software was used to analyze the downstream target molecules of the top 30 miRs in pEVs, and these molecules were mainly related to “cellular movement” and “cardiovascular system development.” **(C)** IPA software was used to analyze the core downstream network that intersected PI3K/AKT signaling and identified 7 key molecules involved in the Akt signaling pathway. **(D)** IPA software and a literature search were used to analyze miRs and their target mRNAs. Among the 30 miRs, 14 miRs targeted the same molecule, PTEN. **(E,F)** Real-time RT-PCR was used to measure miR-92a-3p expression in response to intimal injury or pEV stimulation *in vitro*. The results showed that in response to either pEV stimulation **(E)** or intimal injury **(F)**, miR-92a-3p expression increased significantly. **(G)** In the intimal injury model, FISH was used to measure miR-92a-3p expression *in situ*. The results validated that miR-92a-3p expression levels were significantly upregulated in VSMCs compared with the self-contralateral carotid artery. **(H)** The PTEN 3’UTR has a binding site for miR-92a-3p. **(I)** The qPCR results showed that the miR-92a-3p mimic or inhibitor significantly increased or decreased miR-92a-3p expression in VSMCs, respectively. **(J)** Western blotting indicated that in VSMCs, the miR-92a-3p mimic reduced the protein expression of PTEN, while the miR-92a-3p inhibitor increased PTEN expression compared with that of the control. **(K)** A dual luciferase reporter gene system was used to examine the luciferase activity in wild-type (WT) and mutant PTEN 3’UTRs in negative control and miR-92a-3p mimic-treated HEK-293T cells. miR-92a-3p significantly reduced the luciferase activity of the wild-type PTEN 3’UTR compared with the negative control in three culture replicates. **(L)** Western blotting indicated that in VSMCs, miR-92a-3p mimics increased the protein expression of Col8a1, which was mediated by PTEN. The values are shown as the mean ± SD, *P < 0.05, **P < 0.01 vs. control (*n* = 4 biological replicates).

Recent studies have shown that pEVs can deliver a variety of miRs (Laffont et al., 2013). MiR sequencing was used to identify the top 30 miRs expressed in pEVs secreted from activated platelets (Figure 5A). The functions of the downstream target molecules (82 in total) of these 30 miRs were analyzed by IPA software, and we found that the downstream molecules were mainly related to “cellular movement” and “cardiovascular system development” (Figure 5B and Supplementary Table 5). A total of 41 of the 82 molecules formed a core network structure, of which 7 key molecules were involved in the Akt signaling pathway (Figure 5C and Supplementary Table 6).

Among the 30 miRs, 19 core seed sequences could bind to the 3’UTRs of different target gene mRNAs (Supplementary Table 7). Among the 30 miRs, 14 miRs targeted the same molecule, PTEN, which was the most common of all 7 targeted molecules associated with PI3K/Akt signaling (Figure 5D and Supplementary Table 8). Therefore, miR-92a-3p, which targeted PTEN and was the most highly expressed in pEVs, was selected for follow-up studies.

After pEV stimulation for 12 h *in vitro*, the qPCR results verified that miR-92a-3p expression in VSMCs was significantly upregulated in comparison with that of the control (Figure 5E). To detect whether the increased miR- 92a-3p was produced by VSMCs or was delivered by pEVs to VSMCs, the precursor of miR-92a-3p (pre-miR-92a-3p) in VSMCs was detected. If the miR-92a-3p was produced by VSMCs, pre-miR-92a-3p would increase in VSMCs (Supplementary Figure 6). The results showed that the expression of pre-miR-92a-3p were similar between the control group and pEVs stimulation group, which indicated that the increased miR-92a-3p was transferred by pEVs. Moreover, western blotting showed that PTEN expression in VSMCs was significantly downregulated after pEV stimulation for 24 h (Supplementary Figure 7). *In vivo*, qPCR and FISH results verified that miR-92a-3p expression levels were significantly upregulated in VSMCs 2 weeks after intimal injury surgery in comparison with those of the self-contralateral carotid artery (Figures 5F,G). There was a predicted binding site for miR-92a-3p at the PTEN 3’UTR site 2808-2815 (Figure 5H and Supplementary Table 9), indicating that miR-92a-3p may negatively regulate PTEN protein expression.

Subsequently, miR-92a-3p was overexpressed or knocked down in VSMCs with a specific mimic or inhibitor, respectively (Figure 5I). Western blotting indicated that the miR-92a-3p mimic significantly reduced the protein expression of PTEN, whereas the inhibitor increased PTEN levels compared with those of the respective NC (Figure 5J). These results indicate that miR-92a-3p plays an important role in the regulation of PTEN expression.

Dual luciferase reporter gene analysis was then used to assess the binding and inhibitory capacity of miR-92a-3p for the target sites of the PTEN 3′UTR. Compared with the NC, cotransfection of the miR-92a-3p mimic with the wild-type 3′UTR of PTEN significantly decreased the luciferase activity in HEK-293T cells (Figure 5K). In contrast, there was no significant change when miR-92a-3p was cotransfected with the mutant PTEN 3’UTR compared to the NC (Figures 5H,K).

Then, we evaluated the impact of miR-92a-3p changes on Col8a1. The qPCR and western blot results indicated that the miR-92a-3p mimic significantly increased the mRNA and protein expression of col8a1 (Figure 5L and Supplementary Figure 8). These results indicated that miR-92a-3p negatively regulated the expression of PTEN and subsequently promoted the production of Col8a1 in VSMCs.

### Knockdown of Col8a1 Downregulates Vascular Stiffness

**FIGURE 6:**
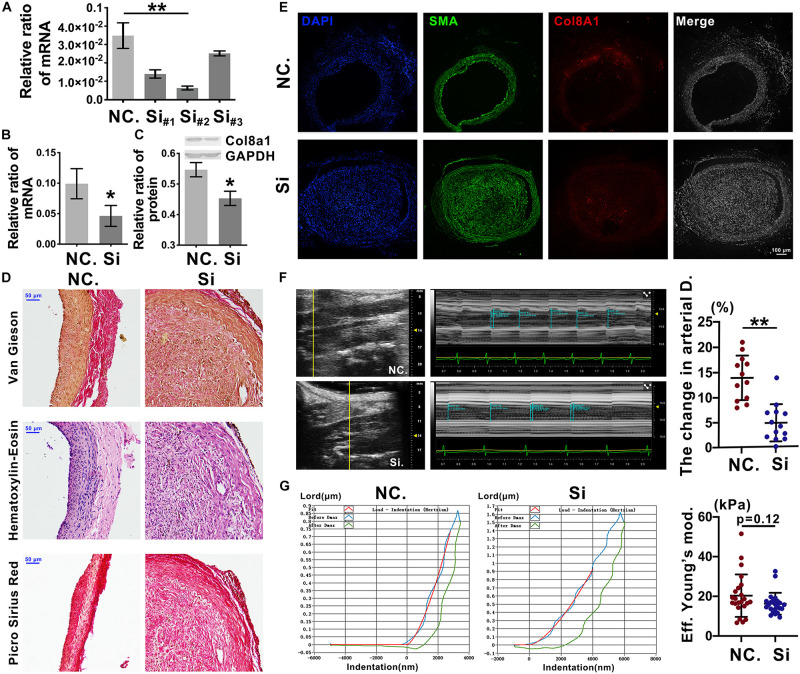
Knockdown of Col8a1 downregulated vascular stiffness. **(A)** Real-time RT-PCR results showed the interference efficiencies of 3 col8a1 siRNA sequences, and si#2 was the most efficient siRNA. **(B,C)** Real-time RT-PCR and western blotting were used to determine the efficiency of si#2 *in vivo*. Compared with the NC, si#2 significantly reduced the mRNA **(B)** and protein **(C)** expression of col8a1. **(D)** Elastin-van Gieson, hematoxylin-eosin (HE), and picrosirius red staining revealed that compared with that of NC administration, neointimal hyperplasia and vascular morphology were thickened after Col8a1 knockdown, but the collagen density was reduced (bar = 50 μm). **(E)** Immunofluorescence was used to examine Col8a1 accumulation in the col8a1 siRNA and control injection groups. Green indicates SMA staining (VSMCs), red indicates Col8a1 staining, and nuclear staining is shown in blue by DAPI (bar = 100 μm). **(F)** Ultrasound imaging was used to measure the change in arterial diameter (%). After col8a1 siRNA injection, at 2 weeks after intimal injury surgery, the change in arterial diameter was significantly increased. **(G)** The Piuma nanoindenter was used to determine vascular stiffness, and the stiffness of the carotid artery decreased from an average of 20.31 kPa to an average of 16.28 kPa compared with that of the NC group. The values are shown as the mean ± SD, *P < 0.05, **P < 0.01 vs. control (*n* = 4 biological replicates).

To further elucidate the effects of Col8a1 on vascular stiffness in the intimal injury model, col8a1 small interfering RNA (siRNA) was injected locally to knockdown Col8a1 expression (Supplementary Figure 9). Three pairs of specific siRNAs were designed, and the most efficient siRNA, si-#2, was identified (Figure 6A and Supplementary Table 1). In intimal injury, the siRNA significantly reduced the mRNA and protein expression of col8a1 compared with that of the NC (Figures 6B,C). Vascular morphological analysis revealed that the vessel wall was thickened, but the collagen density was reduced after col8a1 siRNA injection (Figures 6D,E).

Regarding the mechanical properties of the carotid artery, compared with that of NC injection after intimal injury, siRNA injection significantly decreased the change in arterial diameter (%) at 2 weeks after intimal injury surgery from an average of 13.93% to an average of 4.82% (Figure 6F). Moreover, the stiffness of the carotid artery decreased from an average of 20.31 kPa to an average of 16.28 kPa compared with that of the NC group (Figure 6G). These results suggested that Col8a1 knockdown could alleviate the increase in vascular stiffness in the intimal injury model.

## Discussion

Mechano-homeostasis plays a key role in maintaining the biological functions of the cardiovascular system and the pathological development of cardiovascular disease (Chien, 2007; Haga et al., 2007). A variety of mechanical stresses change, coordinate and control a variety of vascular cells, including ECs and vascular smooth muscle cells (VSMCs) (Tanaka et al., 2019; Zhang et al., 2020; Ngai et al., 2020). Therefore, exploring the changes in the mechanical stress that arterial cells are subjected to is crucial to understanding the mechanobiological mechanisms underlying vascular cell dysfunction. Here, we found that pEVs target VSMCs and effectively promote the production of Col8a1, an important component of the ECM that participates in vascular stiffness.

In the pathogenesis of many cardiovascular diseases, ECM remodeling is closely related to vascular stiffness. Alterations in extracellular matrix composition and arterial geometry result in structural arterial stiffness (Namba et al., 2019). In a murine femoral wire injury model associated with intimal hyperplasia, there is substantial collagen and proteoglycan deposition (Fu et al., 2014), which may cause changes in vascular stiffness. In addition, collagen type I (Lee et al., 2009; Kanshana et al., 2018), type III (Kanshana et al., 2018), type VIII (Plenz et al., 1999) and fibronectin (Yao et al., 2009) are upregulated in different animal intimal injury models. For example, in mechanically injured iliac artery segments in rabbits, collagen types I and III and VSMCs are increased, and there is a reduction in the immunolabeling of macrophage markers (CD68) (Kanshana et al., 2018). In addition, the matrix metalloproteinase (MMP) family, is also essential for arterial stiffening (Liu et al., 2015). MMPs can lead to degradation and remodeling of the arterial ECM and affect arterial stiffening (Liu et al., 2015). In mechanical intimal injury, MMP2 (Tummers et al., 2010) and MMP9 (Cho and Reidy, 2002) are significantly elevated. After acute vascular injury, MMP12 is induced in VSMCs and influences elastin degradation and arterial stiffening (Liu et al., 2015).

Col8a1 is a short chain member of type VIII collagen (Greenhill et al., 2000). In the vessel wall, Col8a1 is mainly located in the subendothelial space, subendothelium and media of arteries, and is secreted by ECs and VSMCs (Plenz et al., 2003). It has been reported that the increased expression of Col8a1 promotes proliferation and migration of ECs and VSMCs (Plenz et al., 2003). At earlier stages of atherogenesis, endothelial derived Col8a1 has been proved to be a key factor in EC proliferation which may play a positive role in endothelial repair and the integrity of neointima (Plenz et al., 1999, 2003). Whereas, VSMC derived Col8a1 mainly takes part in the atherosclerotic plaque development at later stages (Yasuda et al., 2000), and increases VSMC proliferation in ApoE^–/–^ atherosclerotic mice (Yasuda et al., 2000; Lopes et al., 2013). In rat carotid artery balloon injury model, Col8a1secreted by VSMC is overexpressed in the luminal part of carotids after endothelial denudation, and strongly promotes migration of VSMCs (Sibinga et al., 1997). In our study, silencing Col8a1 induced the neointimal hyperplasia which suggested the increased contents of VSMCs, meanwhile, reduced the stiffness of the carotid artery. These studies suggest that although the indiscriminate repression of Col8a1 in all vascular cells could alleviate the increased vascular stiffness, it may also block EC repair which causes excessive proliferation of VSMCs. Whether the VSMC specific knockdown of Col8a1 will be a potential therapeutic target for treating neointimal hyperplasia induced by vascular intima injury and carotid surgery is still unclear and should be further studied in the future.

After vascular intima injury and carotid surgery, the adhesion and aggregation of circulating platelets on the injured intima was an important pathological process (Zaldivia et al., 2017). The activation of platelets and pEV secretion at the intima injury and atherosclerotic plaque could cause VSMC phenotypic switching, migration and proliferation, and subsequent facilitate vascular calcification (Schurgers et al., 2018). Hence, the antiplatelet agents could significantly reduce the risk of carotid stenosis recurrence by reducing platelets deposited at the site of the injured endothelium and vascular inflammation (Dharmakidari et al., 2017; Liu et al., 2018). However, the antiplatelet agent still has its limitations in the treatment of intimal injury. For example, despite the widespread application of drug-eluting stents and anti-platelet therapy, instent restenosis remains a major clinical issue in percutaneous coronary interventions (Waksman and Steinvil, 2016; Liu et al., 2018). In terms of basic research, antiplatelet agent–acetylsalicylic acid (ASA; aspirin), has no significant effect on vascular remodeling in response to denudation injury to rat carotid arteries (Yang et al., 2004). Rosińska’s research also reported that despite the ani-aggregation role of aspirin in platelet, it has no significant effect on microvesicle parameters, including pEVs (Rosińska et al., 2019). Hence, further understanding the platelet and pEVs relative mechanisms in intimal injury may provide novel targets to prevent intimal hyperplasia and vessel or stent occlusion.

In 1967, platelet-derived microparticles (pMPs), which are now called pEVs, were first identified during blood coagulation research and were called platelet dust (Wolf, 1967). We now know that pEVs contain abundant genetic materials and bioactive molecules and transfer these contents from platelets to recipient cells in the circulatory system (Puhm et al., 2020). In our study, pEVs activated PIP_3_/Akt signaling to promote Col8a1 production. Interestingly, several inflammatory factors, oxidative stress-related factors were also changed by pEVs (Supplementary Figure 10). In fact, based on the results reported by Dean et al. (2009), we analyzed 449 proteins present in pEVs and examined their relationship with PIP_3_/Akt signaling. Functional analysis of these molecules by IPA software showed that these 449 molecules were mainly related to “inflammatory response” and “cellular compromise” (Supplementary Figure 11A), and among them, OCRL, ITGB3, and RAP1A. may be involved in the activation of the Akt pathway (Supplementary Figure 11B). The molecular mechanism by which pEVs activate VSMCs is quite complicated and still requires further research.

Many miRs are involved in the development of cardiovascular disease (Wojciechowska et al., 2017). The different miRs, including miR-92a, have different effects in the occurrence and development of cardiovascular diseases. In vascular ECs, up-regulated miR-92a in neointimal lesions caused by wire-induced injury, impairs cell proliferation and migration *via* repressing integrin α5 and sirtuin1, which subsequently reduces eNOS (Daniel et al., 2014). In VSMCs, increased expression of miR-92a, induced by PDGF-BB during the formation of atherosclerosis plaque in mice, promotes cell proliferation and migration via targeting KLF4 (Wang et al., 2019). These results indicate that miR-92a induces the dysfunctions of vascular cells and participates in the pathological process of intimal injury or atherosclerosis. On the other hand, a protective effect of miR-92a on vascular homeostasis was also reported. In older adults and old B6D2F1 mice (an established model of vascular aging), miR-92a expression is reduced which contributes to age-related endothelial dysfunction and increased large artery stiffness (Hazra et al., 2016). In our present work, miR-92a-3p delivered by pEVs targets to PTEN, which then activates the Akt signaling and causes Col8a1 deposition at the intimal injury arteries. These works revealed a varied of functions of miR-92a in different cardiovascular diseases and in different types of cells. This may due to the difference in the pathogenesis of cardiovascular diseases and the complexity of the biological signaling networks caused by miR-92a.

In summary, the current study revealed that during intimal injury, pEVs are located at the injury site, contact VSMCs, and may deliver miR-92a-3p, which in turn represses PTEN and promotes the activation of the Akt pathway and Col8a1 production in VSMCs. Knockdown of Col8a1 expression abrogated the increase in carotid artery stiffness. Therefore, pEVs and the key molecules involved in stiffness modulation may be potential therapeutic targets for neointimal hyperplasia.

## Data Availability Statement

The datasets presented in this study can be found in online repositories. The names of the repository/repositories and accession number(s) can be found below: NCBI Gene Expression Omnibus, accession no: GSE164050.

## Ethics Statement

The animal study was reviewed and approved by Animal Research Committee of Shanghai Jiao Tong University.

## Author Contributions

HB and Y-XQ designed the research. HB, Z-TL, L-HX, T-YS, YH, MB, ZL, Y-JF, YL, and YC performed the research. HB and Z-TL analyzed the data. HB, Z-LJ, X-BG, and Y-XQ wrote the manuscript. All authors contributed to the article and approved the submitted version.

## Conflict of Interest

The authors declare that the research was conducted in the absence of any commercial or financial relationships that could be construed as a potential conflict of interest.

## Footnotes

1 https://biit.cs.ut.ee/clustvis/

2 https://www.qiagenbioinformatics.com

3 https://www.uniprot.org

## Abbreviations

3 ′ UTRs: 3 ′ untranslated regions
Col8a1: Collagen type VIII alpha 1 chain
CS: cyclic stretch
ECM: extracellular matrix
ECs: endothelial cells
HE staining: hematoxylin-eosin staining
IPA: ingenuity pathway analysis
MMP: matrix metalloproteinase
NTA: nanoparticle tracking analysis
OSS: oscillatory shear stress
pEVs: platelet-derived extracellular vesicles
pMPs: platelet-derived microparticles
SD rats: Sprague-Dawley rats
siRNA: small interfering RNA
VSMCs: vascular smooth muscle cells.
