# Supplementary material for: Platelet-Derived Extracellular Vesicles Increase Col8a1 Secretion and Vascular Stiffness in Intimal Injury
Source: Front Cell Dev Biol. 2021 Mar 2;9:641763. doi: 10.3389/fcell.2021.641763 (PMC7960786; doi:10.3389/fcell.2021.641763)
Supplement: Supplementary file 1 [file Data_Sheet_1.docx]

**Supplemental Materials for**

**Bao H, et al. Platelet-derived extracellular vesicles increase Col8a1 secretion and vascular stiffness in intimal injury**

**Supplementary Materials and Methods**

**Hematoxylin-Eosin, Elastin Van Gieson and Sirus Red Stain**

Carotid arteries were fixed in 4% paraformaldehyde, dehydrated in 30% sucrose, and embedded in paraffin (Leica Geosystems 7P1020, Germany). Six μm sections were prepared (Leica Geosystems RM2265, Germany).

Hematoxylin-Eosin staining (HE staining) was used to detect the morphology of artery after intimal injury for 2 wks. The undamaged right carotid artery served as the self-control. Sections were stained with hematoxylin for 8 min, rinsed with distilled water, and immersed into 0.1% hydrochloric acid in 70% ethanol for 5 s. After rinsed with tap water for 5 min, 0.3% ammonium hydroxide was used for 5 min. Sections were stained with eosin for 30 s, and rinsed again with distilled water. Increasing concentrations of ethanol (70%, 90%, 95% and 100%, respectively) were then used for dehydration, which followed by xylene twice. In HE staining, the cell nucleus was stained with hematoxylin into blue, and the cytoplasm was stained with eosin into pink.

Elastin Van Gieson staining was used to visualized the elastic fibers in artery after intimal injury for 2 wks. The undamaged right carotid artery served as the self-control. Sections were stained with Weigert Solution (Fuchsin basic, resorcinol, water, and hydrochloric acid) for 6 h, directly immerged into Differentiation Solution (1% hydrochloric acid alcohol), and then flushed with water. Van Gieson Dye Solution (1% Fuchsinacid aqueous solution and saturated aqueous picric acid solution) was used to restain the sections for 1-2 min (Huang et al., 2017). In Elastin Van Gieson staining, the collagen fibers were stained into bright red, and the remaining connective tissues were stained into yellow.

The Sirus Red was a strongly acidic anionic dye that could react with alkaline collagen. Sections were stained with Picro Sirus Red Solution (Maokangbio, China) for 1 h, and rinsed with distilled water. Sections were stained with Mayer’s Hematoxylin Dye Solution for 8 min, and rinsed with distilled water. Increasing concentrations of ethanol (70%, 90%, 95% and 100%, respectively) were then used for dehydration, which followed by xylene twice. Staining at the cellular level, 4% paraformaldehyde was used to fix VSMCs for 30 min and then followed the above method. In Sirus Red staining, the collagen fibers were stained into red, the nucleus were stained into blue, and other components were stained into yellow.

The microscope (Olympus Corporation IX71, Japan) was used to observe images.

**Transmission electron microscope (TEM) analysis**

Samples were prepared as previously described (Liu et al., 2002). Ultrathin sections were cut with an Ultratome (UC6-FC6, Leica, Germany), counterstained with uranyl acetate and lead citrate, and observed under an electron microscope (Tecnai G2 Spirit, FEI, USA).

**VSMCs culture**

Male Sprague-Dawley rats (160-180 g) were euthanized with intraperitoneal pentobarbitone injection (75 mg/kg). Primary VSMCs were cultured from the carotid artery via an explanting technique. After the removal of the adventitia and endothelium, the media of carotid artery was isolated surgically and minced into small pieces, which were plated onto 25 cm^2^ culture flasks with DMEM (Gibco, USA) containing 10% heat-inactivated FBS (Gibco, Australia) and were incubated at 37°C in a humidified incubator (95% air and 5% CO_2_). The VSMC monolayers were passaged every 2-3 d after trypsinization, and cells in passages 4-7 were used for experiments.

**Preparation of pEVs**

pEVs were isolated following the previous researches (Liu et al., 2006; Laffont et al., 2013). Briefly, whole blood was collected from the abdominal aorta of anesthetized rats into a syringe containing 100 μL·mL^-1^ anticoagulant in 0.5% sodium chloride solution. Platelets were harvested by centrifugation at 1000 g for 10 min and resuspended in HEPES-Tyrode buffer (130 mM NaCl, 3 mM KCl, 0.3 mM Na_2_HPO_4_, 12 mM NaHCO_3_, 20 mM HEPES, 5 mM monohydrate D-glucose, 0.5 mM MgCl_2_, pH 7.4). The resuspended platelets were activated with 1 μL·mL^-1^ collagen I for 1 h and then centrifuged at 2800 g for 15 min to prepare a platelet-free releasate. pEVs were collected from the remaining supernatant by centrifuging at 20500 g for 90 min. NTA were then used to detection the size and number of pEVs.

**Stimulation of VSMCs with pEVs**

The VSMCs, completely attached to the coated glass plate, were incubated with pEVs (10^9^·mL^-1^) for 1 h or 24 h at 37°C with 5% CO_2_. To analyze the adhesion of pEVs to VSMCs, pEVs were labeled with PKH26 (Sigma-Aldrich, USA), and incubated with VSMCs for 1 h. The VSMCs were fixed with 4% paraformaldehyde for 30 min. Then, SMA antibody (1:500, Invitrogen, USA) and DAPI was used to stain. Photographs were taken by confocal microscopy (LV1000; Olympus).

**Detection the adhesion and spreading of VSMCs**

The newly digested and suspended VSMCs were mixed with pre-extracted pEVs (10^9^·mL^-1^) for 5 min, then the mixture was seeded onto an uncoated glass plate for 24 h at 37°C with 5% CO_2_ (Humphries et al., 2009). The VSMCs were fixed with 4% paraformaldehyde for 30 min, and were visualized with a brightfield microscope. Then VSMCs were labeled with SMA antibody (1:500, Invitrogen, USA), and DAPI. Photographs were taken by confocal microscopy (LV1000; Olympus) to calculate the maximum length of microfilament, cell height and area of the VSMCs.

**Quantitative real-time PCR**

Total RNA was isolated with TRIzol Reagent. The isolated RNA was reverse transcribed into complementary DNA using the RT primer for miRs (GenePharma, China) or oligo-dT for mRNA. QPCR was performed using SYBR Green Supermix (TaKaRa, USA) and mRNAs expression were normalized against relative to GAPDH, and miRs to U6 snRNA. The PCR condition were as follows: 95°C for 3 min followed by 40 cycles of 95°C for 15 s and 60°C for 30 s. All primers used for quantitative RT-PCR analysis are listed in Supplementary Table 1. Expression of the different genes was measured by the 2^-ΔC(T)^ method (Schmittgen et al., 2008).

**Western blotting**

Western blotting was performed using antibodies directed against Col8a1 (1:300, Proteintech Group, USA), Col8a2 (1:300, Abcepta, USA), PTEN (1:500, Cell Signaling Technologies), p-Akt (1:500, Cell Signaling Technologies), p-PDK1 (1:500, Cell Signaling Technologies), p-mTOR/mTOR (1:500, Cell Signaling Technologies) and GAPDH (1:1000, Cell Signaling Technologies). After incubation with alkaline phosphatase-conjugated secondary antibodies (1:1,000, Jackson Immunoresearch, USA), the signals were visualized with nitroblue tetrazolium/bromo-chloro-indolyl phosphate (Bio Basic, Canada) and quantified with Image Studio.

**Transfection with miR mimics, miR inhibitor or siRNA**

The sequences of rno-miR-92a-3p mimic, rno-miR-92a-3p inhibitor, col8a1 siRNA or the respective negative controls (NC.) (Gene-Pharma, China) were listed in Supplementary Table 1. VSMCs were seeded onto 6-well cell culture plates at a density of 2×10^5^ cells. After a 24 h incubation to allow for attachment, the cells were transfected with Lipofectamine^TM^ 2000 (Invitrogen, USA) and mimics/ inhibitors/ siRNA according to the manufacturer's instructions. Briefly, 100 nmol of siRNAs and 5 μL of Lipofectamine^TM^ 2000 were diluted in Opti-MEM (Invitrogen, USA) to a final volume of 800 μL. After mixing for 15 min at room temperature, the mixture was added dropwise onto the cells, and the cells were incubated at 37°C in a humidified CO_2_ incubator. Non-silencing siRNA with no known homology to rat genes was synthesized as a negative control (NC).

**Supplemental References**

Dean, W. L., Lee, M. J., Cummins, T. D., Schultz, D. J., & Powell, D. W. (2009). Proteomic and functional characterisation of platelet microparticle size classes. *Thromb Haemost*. 102, 711-718. https://doi.org/10.1160/TH09-04-243

Huang, K., Bao, H., Yan, Z. Q., Wang, L., Zhang, P., Yao, Q. P., et al. (2017). MicroRNA-33 protects against neointimal hyperplasia induced by arterial mechanical stretch in the grafted vein. *Cardiovasc Res.* 113, 488-497. <https://doi.org/10.1093/cvr/cvw257>

Humphries M. J. (2009). Cell adhesion assays. *Methods Mol Biol.* 522, 203-210. <https://doi.org/10.1007/978-1-59745-413-1_14>

Laffont, B., Corduan, A., Plé, H., Duchez, A. C., Cloutier, N., Boilard, E., et al. (2013). Activated platelets can deliver mRNA regulatory Ago2·microRNA complexes to endothelial cells via microparticles. *Blood*. 122, 253–261. <https://doi.org/10.1182/blood-2013-03-492801>

Liu, J., Fitzgerald, M.E., Berndt, M.C., Jackson, C.W., Gartner, T.K. (2006) Bruton tyrosine kinase is essential for botrocetin/VWF-induced signaling and GPIb-dependent thrombus formation in vivo. *Blood.*, 108, 2596-603. doi: 10.1182/blood-2006-01-011817.

Liu, Y.Y., Wong-Riley, M.T., Liu, J.P., Jia, Y., Liu, H.L., Jiao, X.Y., et al. (2002). GABAergic and glycinergic synapses onto neurokinin-1 receptor-immunoreactive neurons in the pre-Bötzinger complex of rats: light and electron microscopic studies. *Eur J Neurosci.* 16, 1058-1066. doi: 10.1046/j.1460-9568.2002.02163.x.

Schmittgen, T. D., and Livak, K. J. (2008). Analyzing real-time PCR data by the comparative C(T) method. *Nat Protoc*. 3, 1101-1108. <https://doi.org/10.1038/nprot.2008.73>

**Supplementary Tables**

**Supplementary Table 1.** The sequences of RNA fragments.

| **gene** | **Forward, 5'-3'** | **Reverse, 5'-3'** |
| --- | --- | --- |
| rno-eln-primer | AGACCTGGGTTTGGACTTTCTC | CGGCCACAAGATTTCCCAAAG |
| rno-col4a1-primer | CGCTGGTGCTGAAGGTTCTG | GGCGTGGGCTTCTTGAACATC |
| rno-col6a3-primer  rno-col16a1-primer | GCACCGAGCATCTAGTTCAACC  ACATTGGTGTTGGCATTGCAGG | GAAGTCCTCACAAGTCCCAGC  AATGCCTTGCTGGCCCATTG |
| rno-tnc-primer | CTCAGCCATCACCAACTGTGC | TACTCATGGCCCTTCCAGTG |
| rno-col12a1-primer | TGCCTGGAGAGAAAGGTGAAAG | ACCTGAGTTTCCTGGACGACC |
| rno-col8a1-primer | AAGAGTGGGCAAGGAAGTGG | CATTCCGTGACCTGGCAAACC |
| rno-col5a3-primer | TCTATCCGCTTCCAAGGGACC | AAAGAGGGTCTTCGCCTGTCC |
| rno-acan-primer | CCCTTGGGCAGAAGAAAGATCG | AGCCTGTGCTTGTAGGTGTTG |
| rno-col8a2-primer | CCCATGCAGAAAGGACCAGTG | GTCCATTGGCAGGATCGGTAG |
| rno-nox1-primer | ATGGCATCCCTTTACTCTGACC | AGGGACCATCCACCTCGATC |
| rno-ccl2-primer | CCTGCTGCTACTCATTCACTGG | TGATCTCACTTGGTTCTGGTCC |
| rno-il6-primer | TGGAGTTCCGTTTCTACCTGG | CCTTAGCCACTCCTTCTGTGAC |
| rno-tnf-primer | CCCACGTCGTAGCAAACCAC | GCAGCCTTGTCCCTTGAAGAG |
| rno-mocs1-primer | TCAGCAGGGACATTGTGGTG | GTGGGTAGAAGTTGGCACAGG |
| rno-mybph-primer | GCGTCACCTAAGCCCAAGATC | CTCCCACCTTTAACCTCCAACC |
| rno-glra4-primer | TGGCTGTATGCCTGCTGTTTG | GAAGCGGCCCTCTCTTATGATG |
| rno-rab6b-primer | CTTTCGACGTGTGGCATCAGC | TCAGAGAACATGGAGCCCATC |
| rno-capn9-primer | TCCACGGCCAGAGAATCAAG | CCAAGGGTTACGGACTCTGATG |
| rno-GAPDH-primer | TGAACTTGCCGTGGGTAGAG | GATGGTGAAGGTCGGTGTGA |
| rno-miR-92a-3p mimics | UAUUGCACUUGUCCCGGCCUG | GGCCGGGACAAGUGCAAUAUU |
| rno-miR-92a-3p inhibitor | CAGGCCGGGACAAGUGCAAUA |  |
| mimics NC | UUCUCCGAACGUGUCACGUTT | ACGUGACACGUUCGGAGAATT |
| Inhibitor NC | CAGUACUUUUGUGUAGUACAA |  |
| col8a1 siRNA-#1 | GCCAGCAAGUACCUCAUAUTT | AUAUGAGGUACUUGCUGGCTT |
| col8a1 siRNA-#2  col8a1 siRNA-#3 | CCCAGUAUAUGAAGGAAAUTT  GAGCCCAUGAUGUACACAUTT | AUUUCCUUCAUAUACUGGGTT  AUGUGUACAUCAUGGGCUCTT |
| siRNA NC | UUCUCCGAACGUGUCACGUTT | ACGUGACACGUUCGGAGAATT |
| rno-miR-92a-3p probe  Negative control probe | Biotin-CAGGCCGGGACAAGTGCAATA  Biotin-CAGTACTTTTGTGTAGTACAA |  |

**Supplementary Table 2.** Genes located as “Extracellular Space” in IPA analysis

| **Symbol** | **Type(s)** | **Symbol** | **Type(s)** |
| --- | --- | --- | --- |
| 1300017J02Rik | other | Ccl6 | cytokine |
| A2M | transporter | Ccl7 | cytokine |
| Acan | other | Ccl9 | cytokine |
| ADAMTS4 | peptidase | CCL17 | cytokine |
| ADAMTS7 | peptidase | CCL21 | cytokine |
| ADAMTS12 | peptidase | CCL22 | cytokine |
| ADAMTS14 | peptidase | CCL3L3 | cytokine |
| ADAMTS16 | other | CELA1 | peptidase |
| ADAMTS17 | other | CFAP44 | other |
| ADAMTS18 | peptidase | CFI | peptidase |
| ADAMTSL1 | other | CGREF1 | other |
| AGR2 | other | CILP2 | other |
| AMH | growth factor | CILP | phosphatase |
| AOAH | enzyme | CMTM3 | cytokine |
| APOA4 | transporter | CMTM7 | cytokine |
| APOA5 | transporter | COL10A1 | other |
| Apoc1 | other | COL11A1 | other |
| APOD | transporter | COL12A1 | other |
| APOE | transporter | COL16A1 | other |
| BANK1 | other | COL19A1 | other |
| BMP4 | growth factor | COL20A1 | other |
| BMP5 | growth factor | COL24A1 | other |
| BMP6 | growth factor | COL2A1 | other |
| BMPER | other | COL4A1 | other |
| BRI3BP | other | COL5A3 | other |
| C2 | peptidase | COL6A3 | other |
| C3 | peptidase | Col6a4 | other |
| C6 | other | COL7A1 | other |
| C16orf54 | other | COL8A1 | other |
| C1QA | other | COL8A2 | other |
| C1QB | other | COL9A1 | other |
| C1QC | other | COMP | other |
| C1QTNF3 | other | CPXM2 | peptidase |
| CCL2 | cytokine | CST7 | other |
| Ccl2 | cytokine | CTHRC1 | other |
| CCL4 | cytokine | CUEDC2 | other |
| CCL5 | cytokine | CXCL6 | cytokine |
| CXCL12 | cytokine | GPX3 | enzyme |
| CXCL13 | cytokine | GREB1L | other |
| CXCL14 | cytokine | GSN | other |
| CYTL1 | cytokine | HMCN1 | other |
| DCHS2 | other | HP | peptidase |
| DCN | other | HPX | transporter |
| DEF6 | other | IGF2 | growth factor |
| DLK1 | other | IGFBP2 | other |
| DLL4 | other | IGFBP3 | other |
| DMP1 | other | IHH | enzyme |
| DOK7 | other | IL12A | cytokine |
| DPT | other | IL17B | cytokine |
| DRC1 | other | IL1B | cytokine |
| EBI3 | cytokine | IL1RN | cytokine |
| ECM1 | transporter | IL36B | cytokine |
| EGFL8 | other | INHBB | growth factor |
| EGFLAM | other | INSL3 | growth factor |
| ELN | other | ITIH3 | other |
| EMCN | other | KCP | other |
| EMID1 | other | KIF19 | enzyme |
| EMILIN3 | other | LAMC3 | other |
| ESM1 | growth factor | LCN2 | transporter |
| F5 | other | LGALS3 | other |
| F10 | peptidase | LIF | cytokine |
| FBLN1 | other | LRG1 | other |
| FBLN7 | other | LTBP4 | growth factor |
| FBN2 | other | LY6G6D | other |
| FCGBP | other | LYZ | enzyme |
| FCN1 | other | MAMDC2 | other |
| Fcna | other | Masp1 | peptidase |
| FGF5 | growth factor | MATN3 | other |
| FGF12 | other | MMP3 | peptidase |
| FGF23 | growth factor | MMP9 | peptidase |
| FITM1 | other | MMP12 | peptidase |
| FRZB | other | MMP14 | peptidase |
| FSTL3 | other | MMP19 | peptidase |
| GDF7 | growth factor | MMRN1 | other |
| GDF11 | growth factor | MOG | other |
| GJC3 | transporter | MPO | enzyme |
| MSLN | other | SERPINE1 | other |
| MYH15 | other | SERPINF2 | other |
| MZB1 | other | SH3TC1 | other |
| NAPSA | peptidase | SHH | peptidase |
| NGF | growth factor | SLAMF8 | other |
| NID2 | other | SLAMF9 | other |
| NPTX1 | other | SLIT2 | other |
| Nradd | cytokine | SMOC2 | other |
| NTN1 | growth factor | SMTN | other |
| NXPH1 | other | SOD3 | enzyme |
| OGN | growth factor | SPARCL1 | other |
| OSM | cytokine | SPON2 | other |
| PAMR1 | peptidase | SPP1 | cytokine |
| PAPLN | other | STAC3 | other |
| PARM1 | other | SUSD2 | other |
| PCOLCE2 | other | TAC3 | other |
| PDGFA | growth factor | TF | transporter |
| PI16 | other | TFPI | other |
| PKHD1L1 | other | TGFB1 | growth factor |
| PLA2G7 | enzyme | TGFBI | other |
| PLA2G2D | enzyme | THBS1 | other |
| PLAU | peptidase | THBS2 | other |
| PLD4 | enzyme | THBS4 | other |
| PODNL1 | other | TIMP3 | other |
| POSTN | other | TIMP4 | other |
| PPM1N | other | TLL1 | peptidase |
| PRG4 | other | TNC | other |
| PRSS12 | peptidase | TNFAIP2 | other |
| PRSS35 | peptidase | TNFSF13 | cytokine |
| PTX3 | other | TNFSF14 | cytokine |
| PXDN | enzyme | TNFSF18 | cytokine |
| RBP4 | other | TNFSF13B | cytokine |
| REG3A | enzyme | TRH | other |
| RPGRIP1 | other | UCN2 | other |
| SBSPON | other | VASH1 | peptidase |
| SCUBE2 | other | VAV3 | cytokine |
| SECTM1 | other | VEGFC | growth factor |
| SEMA3D | other | VTN | other |
| SEMA3E | other | VWA1 | other |
| VWF | other | WNT16 | other |
| WFIKKN2 | other | WNT10A | other |
| WNT11 | other | XCL1 | cytokine |

**Supplementary Table 3.** “Molecular Function” of the “Extracellular Space” molecules (showed in Supplementary Table 2) was analyzed with UniProt and GO, which revealed the molecules involved in “extracellular matrix structural constituent” and “extracellular matrix organization & metalloendopeptidase activity”.

| **AccID** | **log2FC** | **P-value** | **FDR** | **Style** |
| --- | --- | --- | --- | --- |
| Eln# | 1.24657252 | 0.000566152 | 0.004377147 | up |
| Col4a1# | 1.07392953 | 9.59299E-08 | 2.07464E-06 | up |
| Col6a3# | 1.3466444 | 1.81164E-05 | 0.000223325 | up |
| Timp3* | -1.1728821 | 7.52786E-05 | 0.000774826 | down |
| Mmp14* | 1.26508597 | 0.000121073 | 0.00117643 | up |
| Dpt* | 2.01916307 | 1.99027E-12 | 8.9371E-11 | up |
| Dcn* | -1.1663297 | 4.4991E-05 | 0.000494197 | down |
| Thbs2* | 1.68051177 | 1.93422E-07 | 3.90531E-06 | up |
| Col16a1# | 1.96263894 | 1.30788E-43 | 1.2955E-40 | up |
| Tnc# | 4.94958162 | 5.56995E-26 | 1.09061E-23 | up |
| Col12a1# | 3.07411203 | 4.15537E-11 | 1.55149E-09 | up |
| Col8a1# | 2.84760733 | 7.43022E-19 | 7.5372E-17 | up |
| Col5a3# | 1.57414995 | 9.17569E-05 | 0.000921345 | up |
| Fbln1* | -1.2708813 | 0.00038457 | 0.003151227 | down |
| Adamts12* | 1.7309409 | 7.1788E-07 | 1.27112E-05 | up |
| Thbs1* | 2.12299451 | 8.65131E-05 | 0.000874952 | up |
| Cilp* | 3.88092558 | 3.99832E-52 | 6.1207E-49 | up |
| Col11a1* | 3.76703805 | 3.62126E-11 | 1.37649E-09 | up |
| Nid2* | 2.3404489 | 2.67882E-09 | 7.81778E-08 | up |
| Cthrc1* | 1.20122994 | 0.000173294 | 0.001596334 | up |
| Comp* | 3.15209411 | 1.19977E-13 | 6.45463E-12 | up |
| Thbs4* | 3.51094732 | 3.01784E-09 | 8.73151E-08 | up |
| Acan# | 6.2021454 | 1.39535E-19 | 1.47776E-17 | up |
| Cpxm2* | 1.89297039 | 9.05582E-11 | 3.26533E-09 | up |
| Fbln7* | 1.15976873 | 8.47009E-16 | 6.31097E-14 | up |
| Col8a2# | 2.16766478 | 3.0859E-08 | 7.4553E-07 | up |
| Mmp12* | 8.49794291 | 2.82103E-36 | 1.39716E-33 | up |
| Col20a1# | 3.2989213 | 5.42908E-42 | 4.57102E-39 | up |
| PCOLCE2* | -2.1016964 | 4.06805E-14 | 2.35402E-12 | down |
| Adamts7* | 2.47983223 | 1.45782E-18 | 1.43557E-16 | up |
| Col7a1# | 4.13405818 | 2.32287E-30 | 6.86224E-28 | up |
| Mmp19* | 1.46883868 | 1.06502E-14 | 6.59331E-13 | up |
| Fbn2* | 4.13441893 | 6.58418E-15 | 4.19966E-13 | up |
| Emcn* | 1.40805974 | 5.08695E-08 | 1.17194E-06 | up |
| Adamtsl1* | 1.74366681 | 2.83516E-07 | 5.55131E-06 | up |
| Lamc3* | -1.3657096 | 0.000508347 | 0.003990705 | down |
| Adamts17* | 1.58209656 | 1.3806E-09 | 4.18883E-08 | up |
| Adamts14* | 2.27881016 | 1.81806E-15 | 1.27031E-13 | up |
| Adamts4* | 2.58009739 | 1.37035E-10 | 4.75781E-09 | up |
| Tll1* | 1.96999838 | 1.43758E-15 | 1.02574E-13 | up |
| Vtn* | 1.81576548 | 4.05883E-08 | 9.55897E-07 | up |
| Mmp9* | 7.36772852 | 4.26126E-15 | 2.82501E-13 | up |
| Msln* | 3.9270927 | 2.37131E-08 | 5.8378E-07 | up |
| Cilp2* | 5.7342899 | 5.08229E-15 | 3.29157E-13 | up |
| Spon2* | 3.17412995 | 4.79952E-22 | 6.51767E-20 | up |
| Adamts16* | 3.04494728 | 2.94279E-12 | 1.29477E-10 | up |
| Timp4* | -1.2026674 | 0.000913753 | 0.006606561 | down |
| Prg4* | 1.61813912 | 0.000302851 | 0.002565246 | up |
| Ptx3* | 1.87902614 | 0.00024009 | 0.002106758 | up |
| Col24a1# | 2.27223091 | 0.000465911 | 0.003700693 | up |
| Matn3# | 2.86829172 | 9.06011E-06 | 0.000123035 | up |
| Col10a1# | 4.46574499 | 9.82713E-08 | 2.11881E-06 | up |
| Col19a1# | -2.681274 | 1.8802E-10 | 6.38319E-09 | down |
| Mmp3* | 5.1121795 | 8.70451E-05 | 0.000879275 | up |
| Adamts18* | 2.89688408 | 3.31859E-08 | 7.93774E-07 | up |
| Col2a1# | 2.31959045 | 0.00968134 | 0.044922593 | up |
| Emilin3* | 2.86334108 | 1.04494E-06 | 1.77735E-05 | up |
| Col6a4# | 3.21208265 | 1.5363E-05 | 0.000193491 | up |
| Mmp25* | 1.79224628 | 0.004708623 | 0.02526721 | up |
| Col9a1# | -2.0352727 | 0.000175223 | 0.00160882 | down |
| Dchs2# | 2.08899613 | 0.002058005 | 0.012965063 | up |
| Mmp27* | -4.5853641 | 4.02718E-18 | 3.83128E-16 | down |

“#” in the table means “extracellular matrix structural constituent”; “*” in the table means “extracellular matrix organization & metalloendopeptidase activity”.

**Supplementary Table 4.** All molecules correlated with “formation of extracellular matrix” in IPA.

| **Symbol** | **Location** | **Symbol** | **Location** |
| --- | --- | --- | --- |
| AKT1 | Cytoplasm | D-glucose | Other |
| DPYSL2 | Cytoplasm | fenofibrate | Other |
| Erm | Cytoplasm | hyaluronic acid | Other |
| PDCD6IP | Cytoplasm | Mn2^+^ | Other |
| SRC | Cytoplasm | PD 168393 | Other |
| UGCG | Cytoplasm | inhibitor | Other |
| YES1 | Cytoplasm | proteoglycan | Other |
| ADM | Extracellular Space | trichostatin A | Other |
| APOE | Extracellular Space | tyrphostin AG 1478 | Other |
| FN1 | Extracellular Space | Z-LLL-CHO | Other |
| HGF | Extracellular Space | CD44 | Plasma Membrane |
| Ins1 | Extracellular Space | CD9 | Plasma Membrane |
| PTX3 | Extracellular Space | CSF1R | Plasma Membrane |
| TNFAIP6 | Extracellular Space | FYN | Plasma Membrane |
| VCAN | Extracellular Space | ITGB1 | Plasma Membrane |
| CTNNB1 | Nucleus | PLAUR | Plasma Membrane |
| Mmp | Nucleus | ROR2 | Plasma Membrane |
| NR2E1 | Nucleus | SLC9A1 | Plasma Membrane |

**Supplementary Table 5.** The downstream molecules of the top 30 miRs expressed in pEVs were mainly related to “Cellular Movement” and “Cardiovascular System Development”.

| **Symbol** | **Location** | **Symbol** | **Location** |
| --- | --- | --- | --- |
| ADAMTS14 | Extracellular Space | KRAS | Cytoplasm |
| ADAMTS15 | Extracellular Space | LAMC1 | Extracellular Space |
| ADAMTS2 | Extracellular Space | let-7 | Cytoplasm |
| BCL2L11 | Cytoplasm | LOXL2 | Extracellular Space |
| BCL6 | Nucleus | LOXL4 | Extracellular Space |
| BSG | Plasma Membrane | MAP4K4 | Cytoplasm |
| CCKBR | Plasma Membrane | MAPKAP1 | Cytoplasm |
| Ccl9 | Extracellular Space | miR-122b-3p | Cytoplasm |
| CCN2 | Extracellular Space | MKNK1 | Cytoplasm |
| CCND1 | Nucleus | MKNK2 | Cytoplasm |
| COL1A1 | Extracellular Space | NEFM | Plasma Membrane |
| COL1A2 | Extracellular Space | NFIA | Nucleus |
| COL4A1 | Extracellular Space | NID1 | Extracellular Space |
| COL4A2 | Extracellular Space | NID2 | Extracellular Space |
| COL4A5 | Extracellular Space | OIP5-AS1 | Other |
| COL5A1 | Extracellular Space | OSBPL2 | Cytoplasm |
| COL5A2 | Extracellular Space | P3H1 | Nucleus |
| COL8A1 | Extracellular Space | p70 S6k | Cytoplasm |
| CRP | Extracellular Space | PAK1 | Cytoplasm |
| CRTAP | Extracellular Space | PDCD4 | Nucleus |
| Dst | Plasma Membrane | PDGFA | Extracellular Space |
| EIF4E | Cytoplasm | PDGFB | Extracellular Space |
| EPHA2 | Plasma Membrane | PHF6 | Nucleus |
| EZH2 | Nucleus | PIK3R1 | Cytoplasm |
| FBXO32 | Cytoplasm | PLEC | Cytoplasm |
| FBXW7 | Nucleus | PLK1 | Nucleus |
| GRM3 | Plasma Membrane | PLOD2 | Cytoplasm |
| GSK3B | Nucleus | PRC1 | Nucleus |
| HMGA2 | Nucleus | PRDM1 | Nucleus |
| HOXA1 | Nucleus | PTEN | Cytoplasm |
| HOXA11 | Nucleus | PTGS2 | Cytoplasm |
| HPAT5 | Other | RB1 | Nucleus |
| IKZF1 | Nucleus | RECK | Plasma Membrane |
| IL6 | Extracellular Space | SERPINB5 | Extracellular Space |
| IL6R | Plasma Membrane | SPARC | Extracellular Space |
| KMT5A | Nucleus | SPRY1 | Cytoplasm |
| TGFBR1 | Plasma Membrane | Tpm1 | Plasma Membrane |
| TGFBR2 | Plasma Membrane | TRIM63 | Nucleus |
| TIMP3 | Extracellular Space | TRPS1 | Nucleus |
| TLR3 | Plasma Membrane | VCAN | Extracellular Space |
| TOP2A | Nucleus | WEE1 | Nucleus |

**Supplementary Table 6.** Forty one of the 82 molecules (showed in Supplementary Table 5) formed a core network structure in the downstream molecules.

| **Symbol** | **Location** | **Symbol** | **Location** |
| --- | --- | --- | --- |
| BCL2L11 | Cytoplasm | KRAS# | Cytoplasm |
| BCL6 | Nucleus | LAMC1 | Extracellular Space |
| CCND1# | Nucleus | let-7 | Cytoplasm |
| COL1A1 | Extracellular Space | LOXL2 | Extracellular Space |
| COL1A2 | Extracellular Space | NID1 | Extracellular Space |
| COL4A1 | Extracellular Space | p70 S6k# | Cytoplasm |
| COL4A2 | Extracellular Space | PDCD4 | Nucleus |
| COL5A1 | Extracellular Space | PDGFA | Extracellular Space |
| COL5A2 | Extracellular Space | PIK3R1# | Cytoplasm |
| EPHA2 | Plasma Membrane | PLK1 | Nucleus |
| EZH2 | Nucleus | PRC1 | Nucleus |
| GSK3B# | Nucleus | PRDM1 | Nucleus |
| HMGA2 | Nucleus | PTEN# | Cytoplasm |
| HOXA11 | Nucleus | PTGS2 | Cytoplasm |
| HPAT5 | Other | RB1 | Nucleus |
| IKZF1 | Nucleus | TGFBR1 | Plasma Membrane |
| IL6 | Extracellular Space | TGFBR2 | Plasma Membrane |
| IL6R# | Plasma Membrane | TOP2A | Nucleus |
| Tpm1 | Plasma Membrane | WEE1 | Nucleus |
| TRPS1 | Nucleus |  |  |

“#” in the table means “molecules involved in the Akt signal pathway”.

**Supplementary Table 7.** Nineteen core seed sequences among the 30 miRs the top 30 miRs expressed in pEVs.

| **Seed Sequence**  **(5’ to 3’)** | **Synonym(s)** | **Seed Sequence**  **(5’ to 3’)** | **Synonym(s)** |
| --- | --- | --- | --- |
|  | let-7a-5p, let-7i-5p, let-7c-5p, | AGCUUAU | miR-21-5p |
| GAGGUAG | let-7g-5p, let-7d-5p, let-7b-5p, | UCACAUU | miR-23a-3p |
|  | let-7f-5p | UCAAGUA | miR-26a-5p, miR-26b-5p |
| GCAGCAU | miR-103-3p | GUAAACA | miR-30c-5p, miR-30d-5p |
| ACUAUGC | miR-12206-5p |  | miR-30a-5p |
| CACAGUG | miR-128-3p | UGGCCCU | miR-328-3p |
| ACCACAG | miR-140-3p | CCCUGUC | miR-339-5p |
| GAGAUGA | miR-143-3p | ACUAUAC | miR-3596a |
| CAGUGCA | miR-148a-3p, miR-148b-3p | AUGACAC | miR-425-5p |
| UAGACUG | miR-151-3p | GGAAGAC | miR-7a-5p |
| AACGGAA | miR-191-5p | AUUGCAC | miR-92a-3p, miR-25-3p |

**Supplementary Table 8.** The 19 core seed sequences (showed in Supplementary Table 7) that bind to different target gene mRNA 3'UTRs.

| **From Molecule(s)** | **To Molecule(s)** | **Resource** |
| --- | --- | --- |
| let-7a-5p | CCND1 | IPA |
| let-7a-5p | CCND1 | IPA |
| miR-143-3p | KRAS | IPA |
| miR-143-3p | KRAS | IPA |
| miR-21-5p | PIK3R1 | IPA |
| miR-21-5p | PTEN | IPA |
| miR-21-5p | PTEN | IPA |
| miR-21-5p | IL6R | IPA |
| miR-21-5p | PIK3R1 | IPA |
| miR-21-5p | PTEN | IPA |
| miR-23a-3p | IL6R | IPA  IPA |
| miR-23a-3p | IL6R |  |
| miR-26a-5p | PTEN | IPA |
| miR-26a-5p | GSK3B | IPA |
| miR-26a-5p | GSK3B | IPA |
| miR-26a-5p | PTEN | IPA |
| miR-7a-5p | p70 S6k | IPA |
| miR-7a-5p | p70 S6k | IPA |
| miR-92a-3p | PTEN | IPA |
| miR-92a-3p | CCND1 | IPA |
| miR-92a-3p | PTEN | IPA |
| miR-148a-3p | PTEN | doi: 10.1016/j.ijpara.2019.08.002. |
| miR-103-3p | PTEN | doi: 10.26355/eurrev_201812_16625. |
| miR-23a-3p | PTEN | doi: 10.1186/2047-9158-3-4. |
| miR-425-5p | PTEN | doi: 10.18632/oncotarget.15958. |
| miR-328-3p | PTEN | doi: 10.26355/eurrev_201810_16043. |
| miR-7a-5p | PTEN | doi: 10.1016/j.jaut.2020.102440. |
| miR-128-3p | PTEN | doi: 10.1007/s13277-017-5487-6. |
| miR-140-3p | PTEN | doi: 10.1016/j.ejphar.2019.03.034. |
| let-7a-5p | KRAS | doi: 10.1002/cam4.279. |
| miR-140-3p | CCND1 | doi: 10.26355/eurrev_201912_19678. |
| miR-143-3p | CCND1 | doi: 10.1177/1010428317711312. |
| miR-23a-3p | CCND1 | doi: 10.1158/0008-5472.CAN-17-1262. |
| miR-425-5p | CCND1 | doi: 10.1038/leu.2012.302. |
| miR-7a-5p | CCND1 | doi: 10.1049/iet-syb.2019.0025. |
| miR-21-5p | GSK3B | doi: 10.1074/jbc.M116.772392 |
| miR-7a-5p | GSK3B | doi: 10.1049/iet-syb.2019.0025. |
| miR-30c-5p | IL6R | doi: 10.1590/1414-431X20176246. |

**Supplementary Table 9.** The sequences of synthesized PTEN 3’UTR (wild type, WT) and mutated PTEN 3’UTR for dual luciferase reporter assay. The differences between the WT and the mutated are shown in red.

| **Name** | **Sequence** |
| --- | --- |
| PTEN WT | TCACCTTTAAGAAGTCTTACAGTCGGGCCCCGTACATCCCAAGTCCTCTGTAATTCCTCTTGGACATTTTTTGCCATAATTGTCAAAGGGTAGTTGAATTAAATAGCGTCACCATTCTTTGCTGTGGCACAGGTTCTAAACTTAAGTGGAGTTTACCGGCAGCATCAAATGTTTCAGCTTTAAAAATAAAAGTAGGTTACAAGTTCCATGTTTAGTTTTAGAAAATTT**GTGCAATA**TGTGGATAACGACGGCTGTGGTTGCCACAAGTGCCTCGTTTGCCTTTAAATACTGTTAATGTGTCCTGCATGCAGGCGGAAGGGGTGGGTCTGTGCACTAAACCGGGCGCTTGGACTCTAGTATTTGGCAGAGTTGACTCCTACCTGCCAGTTCA |
| PTEN mutated | TCACCTTTAAGAAGTCTTACAGTCGGGCCCCGTACATCCCAAGTCCTCTGTAATTCCTCTTGGACATTTTTTGCCATAATTGTCAAAGGGTAGTTGAATTAAATAGCGTCACCATTCTTTGCTGTGGCACAGGTTCTAAACTTAAGTGGAGTTTACCGGCAGCATCAAATGTTTCAGCTTTAAAAATAAAAGTAGGTTACAAGTTCCATGTTTAGTTTTAGAAAATTT**CACGTTAT**TGTGGATAACGACGGCTGTGGTTGCCACAAGTGCCTCGTTTGCCTTTAAATACTGTTAATGTGTCCTGCATGCAGGCGGAAGGGGTGGGTCTGTGCACTAAACCGGGCGCTTGGACTCTAGTATTTGGCAGAGTTGACTCCTACCTGCCAGTTCA |

**Supplementary Figures**


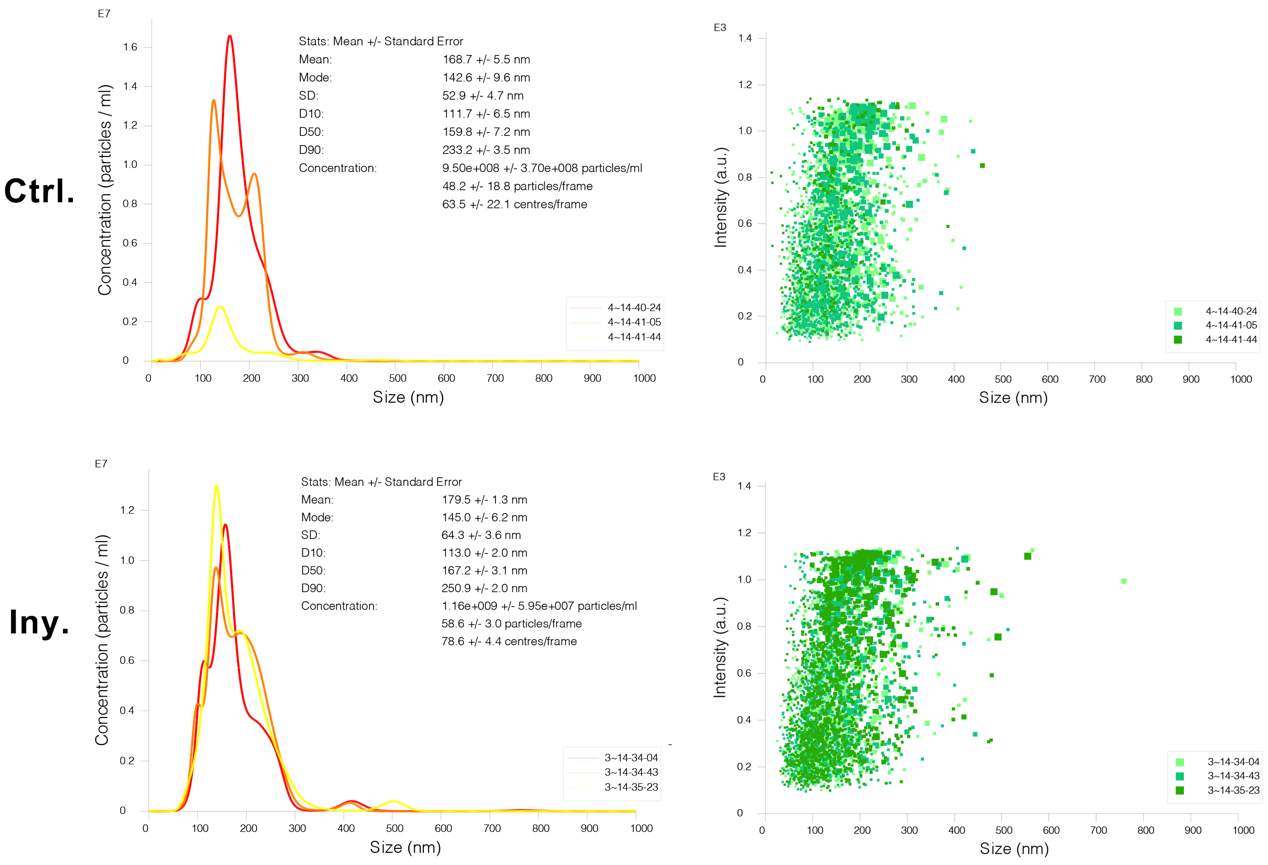


**Supplementary Fig. 1**. Nanoparticle tracking analysis (NTA) was used to examine the concentration and size of the circulating EVs in the control group and intimal injury group.

***
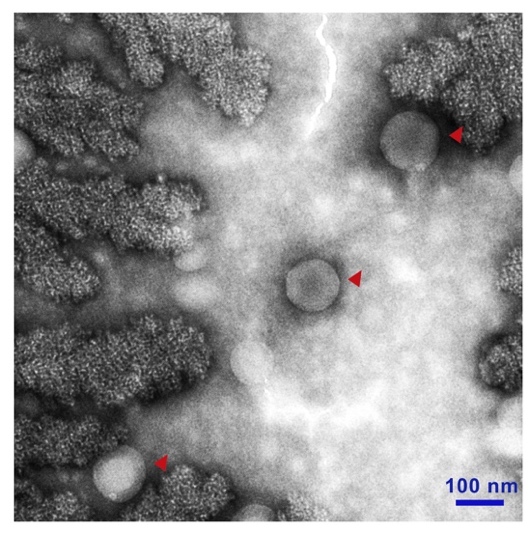
***

**Supplementary Fig. 2.** Transmission electron microscopy (TEM) was used to detect the isolated pEVs. The red arrow indicates the pEVs. Scale bar = 100 nm.


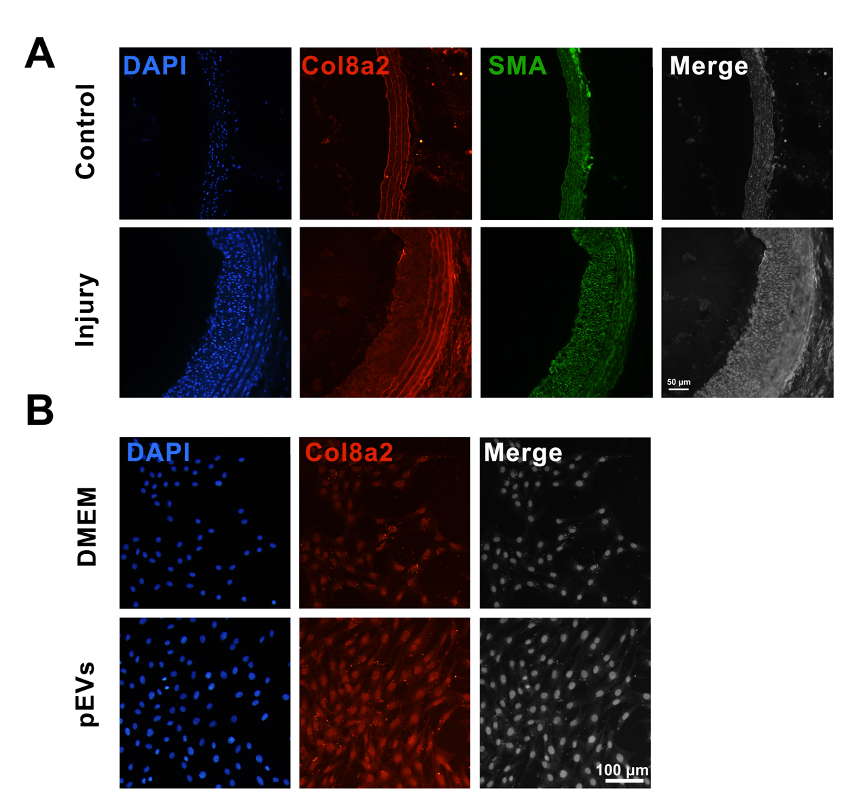


**Supplemental Fig. 3. Immunofluorescence staining was used to detect the expression of Col8a2 in intimal injury *in vivo* or pEVs stimulated VSMCs *in vitro*.** The results showed that the fluorescence intensity of Col8a2 increased significantly in intimal injury **(A)** and VSMCs stimulated with pEVs **(B)**, while most fluorescence signals were located in the nucleus. Green was SMA staining (VSMCs), red was Col8a2 staining, and blue was nuclei staining by DAPI (A: Bar = 50 μm, B: Bar = 100 μm).


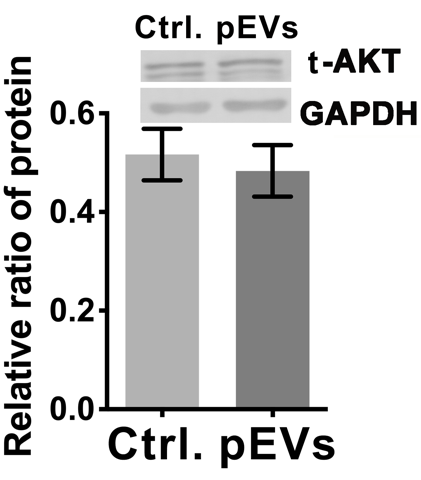


**Supplemental Fig. 4.** Western blotting was used to measure the expression of total Akt in VSMCs. The values are shown as the mean ± SD (n = 4 biological replicates).


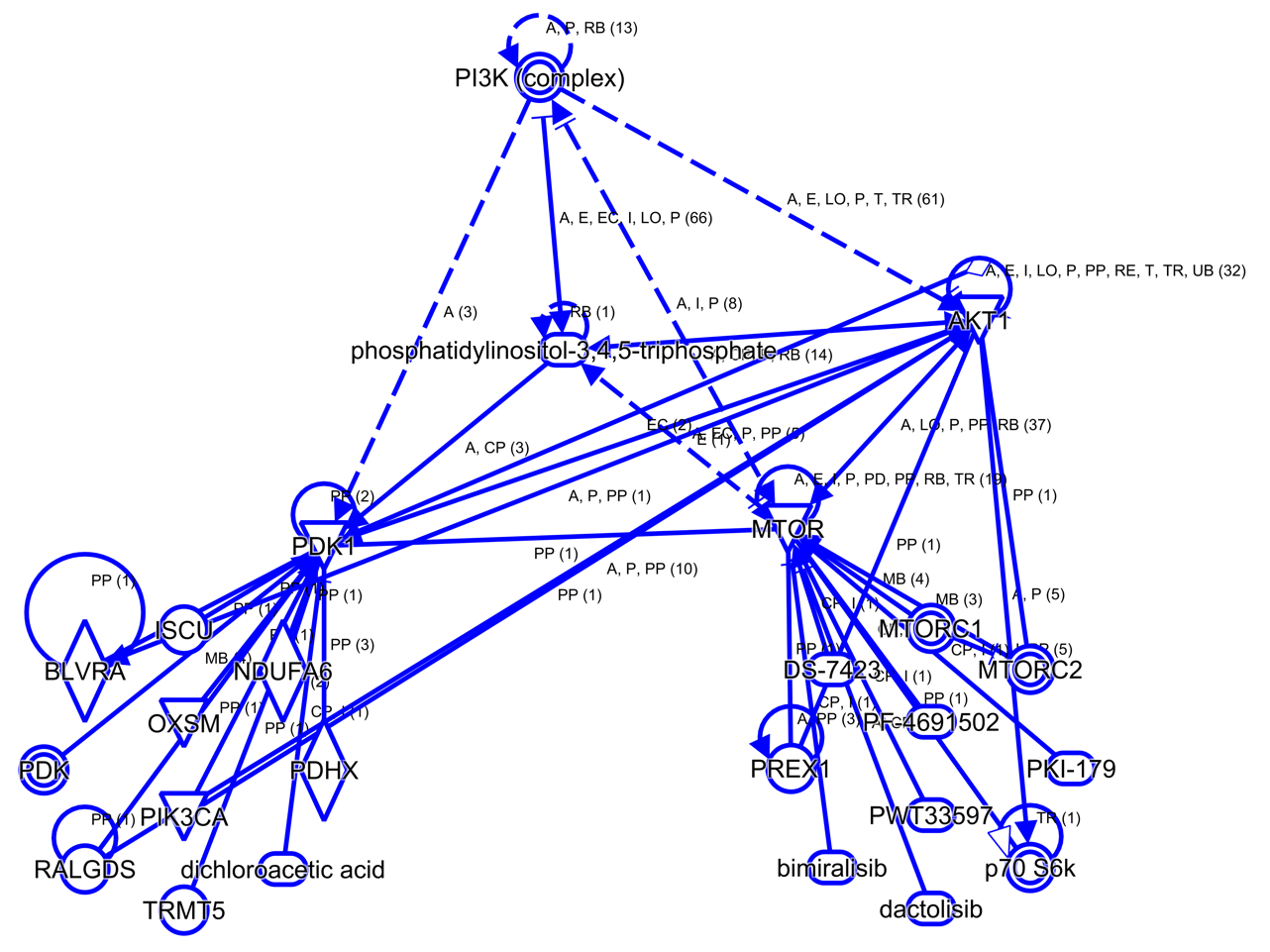


**Supplemental Fig. 5.** IPA software was used to analyze related molecules with PI3K, PIP3, Akt, PDK1 and mTOR.


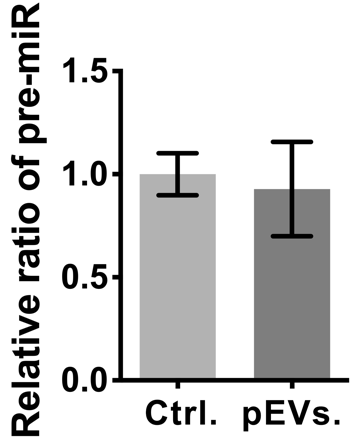


**Supplemental Fig. 6.** Real‐time RT-PCR was used to measure the expression of pre-miR-92a-3p in response to pEV stimulation *in vitro*. The values are shown as the mean ± SD (n = 4 biological replicates).

**
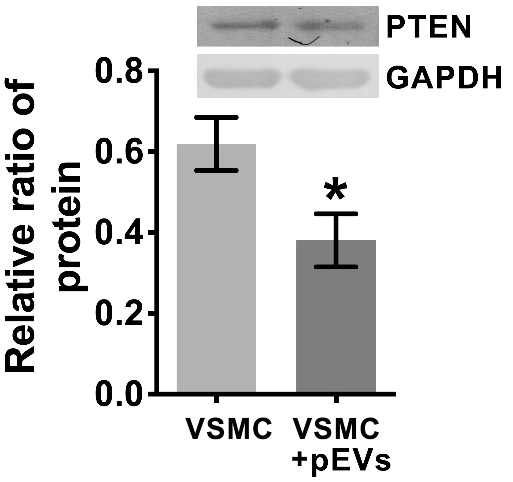
**

**Supplemental Fig. 7. Western blotting was used to detect the expression of PTEN in VSMCs.** PTEN was significantly repressed after pEVs stimulation for 24 h. Values were shown as the mean ± SD, * *P* < 0.05 *vs.* control (n = 4 biological replicates).


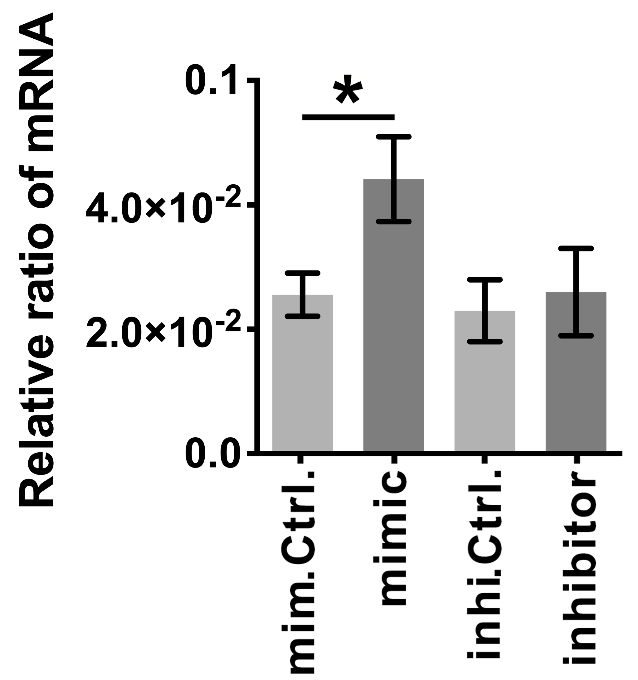


**Supplemental Fig. 8. QPCR was used to detect the mRNA expression of col8a1 in VSMCs.** In VSMCs, miR-92a-3p mimics increased the mRNA expression of col8a1. Values were shown as the mean ± SD, * *P* < 0.05 *vs.* control (n = 4 biological replicates).


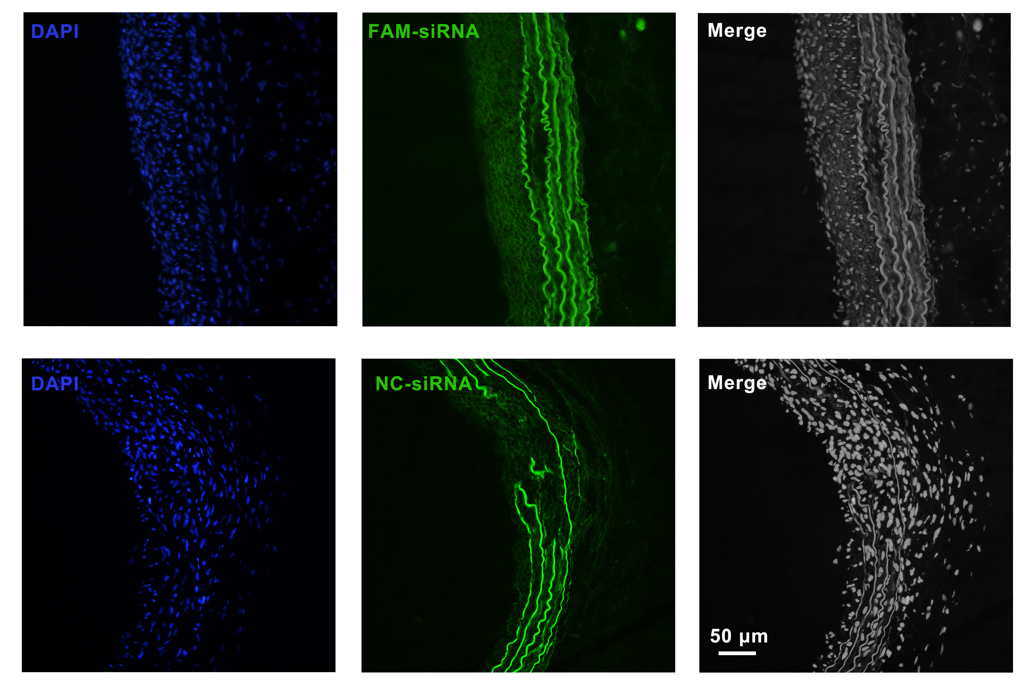


**Supplemental Fig. 9. Col8a1 small interference RNA (siRNA) was injected after intimal injury to knockdown Col8a1.** Green was si-RNA staining (FAM-siRNA) and nuclei staining was shown in blue by DAPI (Bar = 50 μm).

***
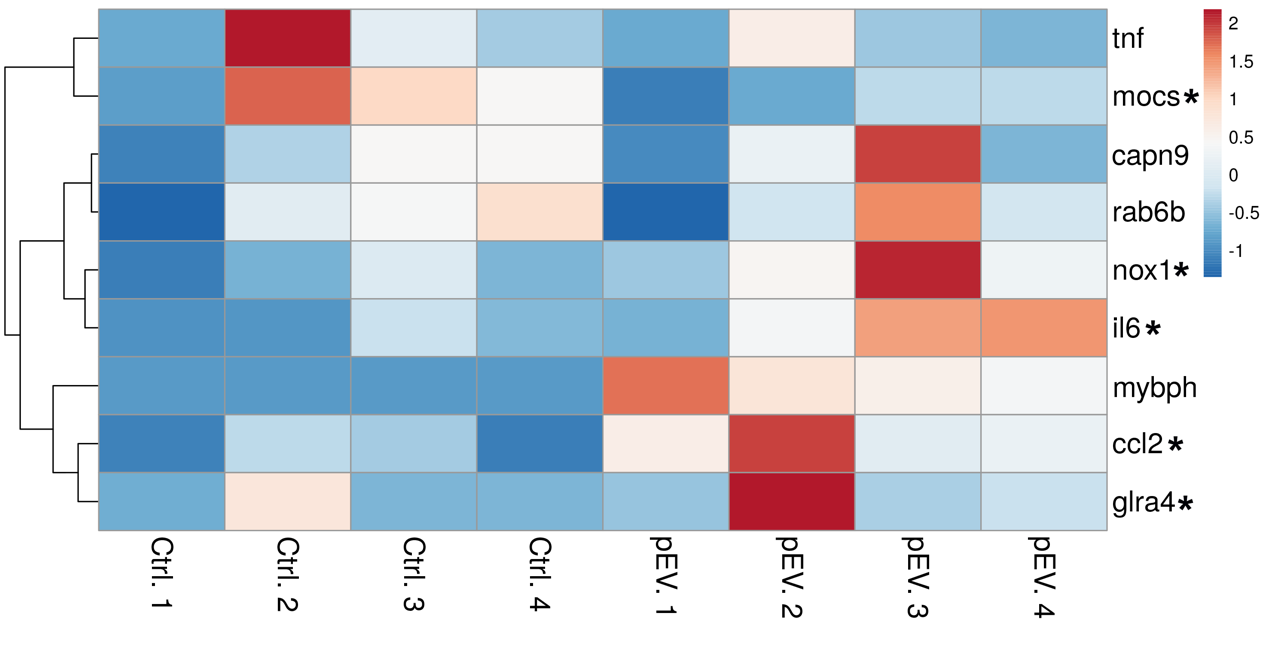
***

**Supplemental Fig. 10.** Real‐time RT-PCR was used to measure the expressions of 9 molecules that participate in inflammatory response, oxidative stress and arterial stiffness. The pEV stimulation group was compared with the control group. The values are shown as * *P* < 0.05 *vs.* control (n = 4 biological replicates).


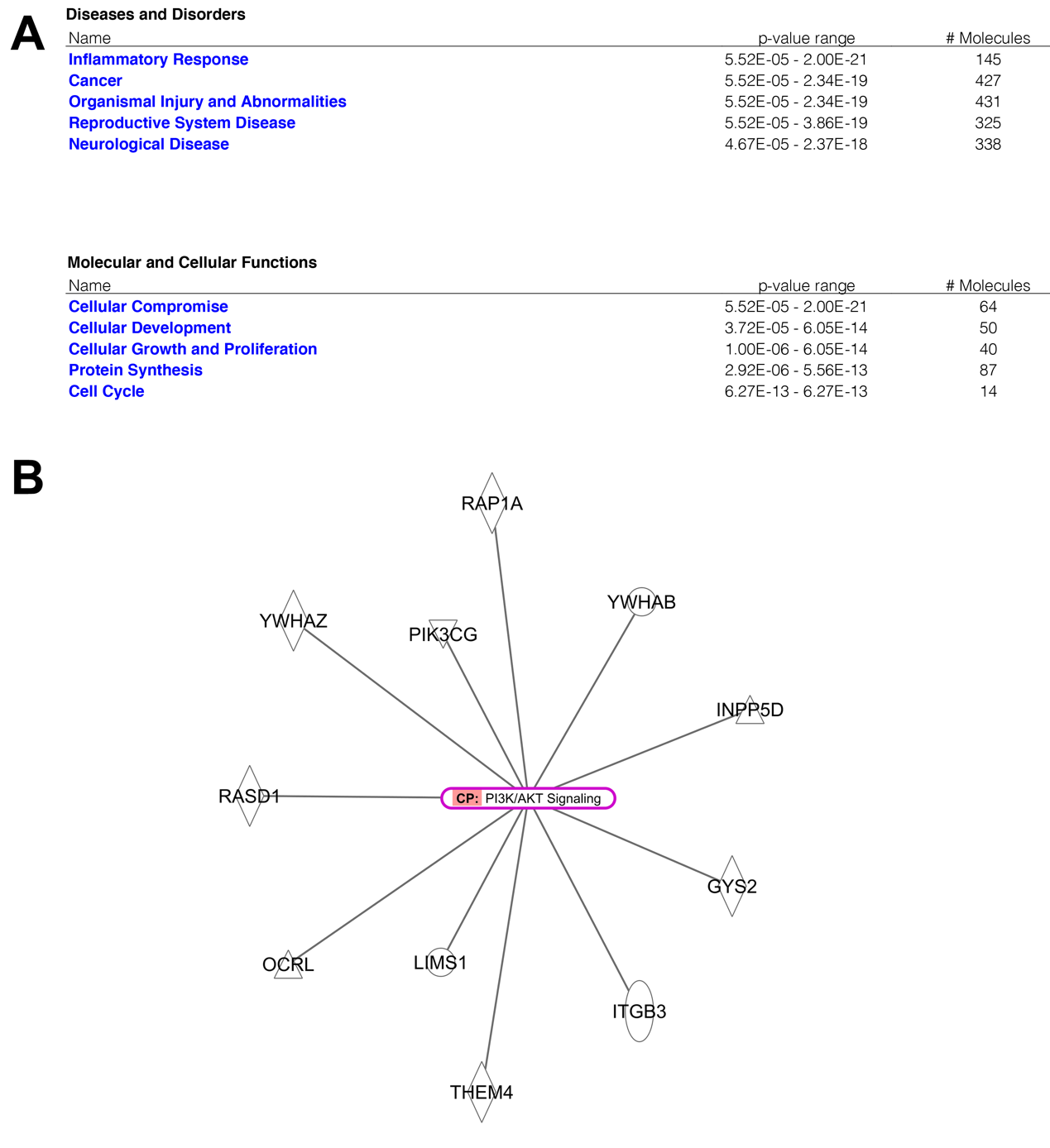


**Supplemental Fig. 11.** IPA software was used to analyze the function **(A)** and molecules involved in PI3K/Akt signaling **(B)** based on the proteomic datas in pEVs (Dean et al., 2009).
